# Fhit Deficiency-Induced Global Genome Instability Promotes Mutation and Clonal Expansion

**DOI:** 10.1371/journal.pone.0080730

**Published:** 2013-11-14

**Authors:** Satoshi Miuma, Joshua C. Saldivar, Jenna R. Karras, Catherine E. Waters, Carolyn A. Paisie, Yao Wang, Victor Jin, Jin Sun, Teresa Druck, Jie Zhang, Kay Huebner

**Affiliations:** 1 Department of Molecular Virology, Immunology and Medical Genetics, Ohio State University Wexner Medical Center, Columbus, Ohio, United States of America; 2 Department of Biomedical Informatics, Ohio State University Wexner Medical Center, Columbus, Ohio, United States of America; University of North Carolina School of Medicine, United States of America

## Abstract

Loss of Fhit expression, encoded at chromosome fragile site FRA3B, leads to increased replication stress, genome instability and accumulation of genetic alterations. We have proposed that Fhit is a genome ‘caretaker’ whose loss initiates genome instability in preneoplastic lesions. We have characterized allele copy number alterations and expression changes observed in Fhit-deficient cells in conjunction with alterations in cellular proliferation and exome mutations, using cells from mouse embryo fibroblasts (MEFs), mouse kidney, early and late after establishment in culture, and in response to carcinogen treatment. *Fhit* ^*-/-*^ MEFs escape senescence to become immortal more rapidly than *Fhit* ^*+/+*^ MEFs; *-/*- MEFs and kidney cultures show allele losses and gains, while +/+ derived cells show few genomic alterations. Striking alterations in expression of p53, p21, Mcl1 and active caspase 3 occurred in mouse kidney -/- cells during progressive tissue culture passage. To define genomic changes associated with preneoplastic changes *in vivo*, exome DNAs were sequenced for +/+ and -/- liver tissue after treatment of mice with the carcinogen, 7,12-dimethylbenz[a]anthracene, and for +/+ and -/- kidney cells treated *in vitro* with this carcinogen. The -/- exome DNAs, in comparison with +/+ DNA, showed small insertions, deletions and point mutations in more genes, some likely related to preneoplastic changes. Thus, Fhit loss provides a ‘mutator’ phenotype, a cellular environment in which mild genome instability permits clonal expansion, through proliferative advantage and escape from apoptosis, in response to pressures to survive.

## Introduction

 In hereditary cancers, genomic instability resulting from mutations in DNA repair genes, known as caretaker genes, drives cancer development, but sequencing of many non-familial cancers has not detected frequent mutations in DNA repair genes. Thus, for sporadic cancers the molecular basis of genomic instability is not known. A prevailing view for the development of genome instability in sporadic cancers is that it is due to oncogene activation at some point during cancer development. According to this view, supported mainly by oncogene overexpression experiments, the mutation patterns of specific tumor suppressor genes, such as *TP53*, support the oncogene-induced DNA replication stress model, which attributes genomic instability and suppressor gene mutations to oncogene-induced DNA damage [1].

 It has been known for years that common chromosome fragile loci, that are expressed as chromatid gaps or breaks in blood cells of all individuals under conditions of mild replication stress [2], are involved in chromosome deletions in early stages of preneoplasia [3-5] before oncogene activation. These deletions, seen in developing cancers as the most frequent genetic alterations, have been considered passenger events rather than drivers [6] of clonal expansion. The human Fhit protein is encoded by the *FHIT/*FRA3B locus [7], the most active fragile locus in human lymphoblasts, though not the most fragile region in cells derived from other tissues [8]. Fhit expression is lost or reduced in many precancerous lesions and in >50% of cancers of humans [9]. Furthermore, *Fhit* knockout (*Fhit* ^*-/-*^) mice are highly susceptible to carcinogen-induction of tumors and *FHIT* replacement in these tumors, by gene therapy, induced apoptosis and significantly reduced tumor burden [10-12]. Numerous reports have confirmed that the *FHIT* gene is a preferential target of allelic deletion and that Fhit inactivation has roles in initiation, development and progression of cancers ([13] for review), and we have recently reported that Fhit protein deficiency causes reduced expression of thymidine kinase, subsequent dTTP imbalance, impaired DNA replication fork progression, and spontaneous DNA breaks that are transmitted to daughter cells, leading to genome instability [14]. Genomic instability is observed in human precancerous lesions, and concurrent loss of Fhit expression has been detected in precancerous lesions, suggesting that, due to fragile site susceptibility to replication fork stress, Fhit loss is among the earliest changes to occur in the preneoplastic process [4,5]. We have concluded that loss of Fhit, a genome ‘caretaker’, initiates the onset of genomic instability in precancerous lesions that drives tumorigenesis and links common fragile site instability to genomic instability and cancer development. Following our finding that loss of Fhit expression leads to accumulation of DNA damage in cells established from Fhit^-/-^ tissues [14], the goal of the current research was to illustrate the consequences of loss of Fhit caretaker function by demonstrating the ‘mutator’ phenotype of Fhit-deficient cells and tissues*.*

## Materials and Methods

### Ethics Statement

 The experiments involving isolation of mouse tissues for DNA analysis and for establishment of cell lines was carried out in strict accordance with the recommendations in the Guide for the Care and Use of Laboratory Animals of the National Institutes of Health. The protocols for these experiments were approved by The Ohio State University Institutional Animal Care and Use Committee (IACUC, Protocol Numbers: 2007A0174-R2 and 2008A0151-R1).  All efforts were made to minimize suffering as outlined in the protocols and approved by the IACUC.

### Cell lines, mouse tissues and reagents

 We previously established mouse embryo fibroblast (MEF)-derived cell lines from 3 *Fhit* ^*+/+*^ and 3 *Fhit* ^*-/-*^ embryos for each genotype, showed that -/- cell lines became immortalized at early tissue culture passage and exhibited Copy Number Variations (CNVs) [14]. Since most human cancers derive from epithelial cells of major organs, we have also established epithelial cell lines from +/+ and -/- baby mouse kidney tissue, cloned lines from these cultures, compared proliferation characteristics and examined the effect of carcinogen treatment on +/+ and -/- cells. To define consequences of the Fhit loss-induced genome instability *in vitro*, we have examined CNVs and mRNA expression in these mouse cell models. To examine *in vivo* effects of genome instability in *-/*- mouse tissue, we have treated +/+ and -/- mice with 7,12-Dimethylbenz[a]anthracene (DMBA, Sigma Aldrich), a carcinogen known to induce mutations in mouse liver [15], and have compared genome-wide exome sequences of +/+ and -/- liver DNAs at 1 and 4 weeks post DMBA injection.

*Fhit* ^*+/+*^ and *Fhit* ^*-/-*^ mouse kidney cells from C57Bl/B6 background mice were cultured in MEM with 10% FBS and 100 μg/ml gentamicin. At passage (P)15, cells were plated at a low density (100 cells per 100 mm culture dish). After 10-12 days, 8 randomly chosen colonies were isolated and designated +/+ clones 1-8 and -/- clones 1-8. *Fhit* ^*-/-*^ cells that survived carcinogen treatment were established after exposure of *+/+* and *-/*- mouse kidney cells to DMBA at P20; 1 x 10^5^ cells from growing cultures were seeded in 60 mm dishes in medium including 20 µM DMBA; 3 days after seeding, the medium was replaced with normal medium and cells were cultivated. Two weeks later, no colonies were observed in *+/+* mouse kidney dishes, whereas 13 colonies were observed among -/- mouse kidney dishes. The colonies were reseeded and cells expanded for further analyses (*-/*- DMBA-surviving cells). 

### Western blot analysis

 Cells were lysed with RIPA buffer (Thermo Scientific) supplemented with Halt Protease Cocktail Inhibitors (Thermo Scientific). Proteins were separated by SDS gel electrophoresis, transferred to nylon membranes and immunoblotted with antisera against murine p21 (F-5) (Santa Cruz), p53 (1C12), Survivin (71G4B7), MCL-1 (D35A5), Cleaved-Caspase 3 (Asp 175), Caspase 3, PARP (Cell Signaling), Fhit [11,14] and GAPDH (Calbiochem). 

### Copy number variation analysis

 Genomic DNA was isolated from *+/+* and *-/*- mouse kidney cells at P4, *+/+* and *-/*- cloned cells (n=3 lines), and *+/+* and *-/*- MEFs at tissue culture P3 and P25 (n = 3 lines) using DNeasy Blood and Tissue isolation kit (Qiagen). Genomic DNA was also isolated from *+/+* and *-/*- B6 weanling tail tissue. Genomic DNA samples were analyzed for CNVs at Jackson Labs using the Affymetrix Mouse Diversity Genotype Array. The B6 *+/+* tail DNA served as reference DNA. This array simultaneously assays 623,124 single nucleotide polymorphisms of 12 inbred mouse strains and more than 900,000 invariant genomic regions in the mouse genome [16]. After analysis of the insertion/deletion data was completed, the single nucleotide polymorphism (SNP) data was used to confirm that deletions and gains were indeed due to B6 allele losses and gains rather than to known germline deletions or to retention of 129svJ alleles. In the results section, CNV loci are classified ‘Loci not known to be fragile’ or ‘Loci known to be fragile’ referring to the reports of Djalali et al [17] and Hosseini et al [18]. 

### Colony formation assay

 For colony formation assays, cells were plated at varying densities into 100 mm culture dishes, grown for 14-21 days. Cells were then fixed and stained with crystal violet and colony numbers counted. For clonogenicity assays, cells were treated with 5 or 20 µM DMBA 24 hr before plating. After DMBA exposure, cells were washed with PBS and growth medium; 3 days later, the surviving cells were replated (2 x 10^5^ cells per 100 mm dish) in growth medium and cultured for 3 wks. Cells were then fixed, stained and colonies counted; mutation frequencies were calculated as average colony formation efficiency.

### Statistical Analyses

 Results of clonogenicity experiments were expressed as average values of three or four measurements. Error bars for all experiments represent one standard deviation around the mean. P values were determined by Student’s two-sided t test in comparisons of results from experiments on Fhit positive and negative cells.

### ß-galactosidase staining for detection of senescent cells

 Senescent cells were detected by ß-Galactosidase staining [19] using the Senescence ß-Galactosidase Staining Kit (Cell Signaling) and recommended protocol. Stained cells were observed and enumerated by light and phase-contrast microscopy.

### Gene expression analysis

 Total RNA was isolated from *+/+* and *-/*- mouse kidney cells at P4 and P15 (n=1 for the kidney cultures) and *+/+* and *-/*- MEFs at P3 and P27 (n=3 for the MEF cultures [14]) using the RNeasy Mini Kit (Qiagen) according to the manufacturer’s instructions. cDNA was synthesized using RT^2^ First Strand kit (Qiagen) and gene expression was examined by RT² Profiler PCR Array (Mouse Cellular Senescence PCR Array) according to the user manual instructions. Data was analyzed by Web-Based PCR Array Data Analysis (Qiagen). Genes differentially regulated in Fhit^-/-^ cells *vs Fhit* ^*+/+*^ cells were reported as relative fold-changes. Relative fold-changes, 2^(-ΔΔC_t_), were calculated by dividing the normalized gene expression, 2^-ΔC_t_, in the test sample by the normalized gene expression, 2^-ΔC_t_, in the control sample. Specifically for the samples tested, relative fold-changes in the genes were calculated by comparing 2^-^ΔC_t_ values in *+/+* mouse kidney cells at P4 compared to *+/+* and *-/*- mouse kidney cells at P15. Fold-changes were also reported for MEFs, comparing 2^-^ΔC_t_ values for *+/+* tissue culture at P3 with and *-/*- MEFs at tissue culture at P27. 

### *Trp53* RT-PCR and Sequencing

 Total RNA was extracted and cDNA synthesis was performed for 15 min at 42°C using 1 µg of total RNA as template, RT primer and quantitect reverse transcriptase (Qiagen Quantitect Reverse Transcription Kit, USA). PCR was then performed using *Trp53* gene specific primers: forward 5’ TGCTCACCCTGGCTAAAGTTCTGT 3’ and reverse 5’ ATGCAGACAGGCTTTGCAGAATGG 3’. Per reaction, 1 U of Platinum PCR SuperMix High Fidelity Taq polymerase was used and the cycling conditions were: pre-incubation for 5 min at 95°C, 30 cycles of 15 s at 95°C, 30 s at 54°C and 2 min at 68°C and final incubation at 4°C. PCR product quality was analyzed on a 0.8% agarose gel and purified for sequencing analysis *via* Qiagen QIAquick PCR Purification Kit. Sequencing samples containing 6.4 pmol of primer and 45 ng of template DNA were brought to a total of 12 µl with DNAse free water and sent for sequencing to the OSUCCC Nucleic Acid Shared Resource. Sequencing primers for the *Trp53* gene were: forward 5’ TGCTCACCCTGGCTAAAGTTCTGT 3’; reverse 5’ ATGCAGACAGGCTTTGCAGAATGG 3’; a second forward 5’ TTACCAGGGCAACTATGGCTTCCA 3’; second reverse 5’ TTCGAGATGTTCCGGGAGCTGAAT3’. 

### Transfection

 The mammalian expression vectors, pRcCMV-*FHIT*, pRcCMV-empty vector, pRcCMV-WT *TP53* or pRcCMV-mouse *Trp53* A135P, were transfected into mouse kidney cells using Lipofectamine 2000 (Invitrogen) reagent. Mouse kidney cells were plated on 60 mm^2^ dishes and cultured to 60-80% confluence. Transfections were carried out with 1 µg plasmid DNA and 2 µl Lipofectamine diluted in Opti-MEM (Gibco) and incubated for 30 min at room temperature. Cells were washed in Opti-MEM, overlaid with the plasmid DNA/Lipofectamine solution and incubated overnight at 37° C. To optimize *FHIT* plasmid transfections, 1 µg DNA was mixed with either 2 or 4 µl Lipofectamine. Cell lysates were collected 24 h post-transfection for western blot analysis.

### Exome sequencing of genomic DNAs of mouse liver tissue and mouse kidney cell lines

 Genome DNA from kidney cells or liver tissue of the following genotypes was prepared for exome sequencing: wild-type (*Fhit* ^*+/+*^) C57BL/6 (B6) and Fhit^−/−^ on B6 background. B6 mice were purchased from the Jackson Laboratory (Bar Harbor, ME). Fhit^−/−^ mice were generated as described [10,11]. Mice were treated at 10-14 days of age with a single intraperitoneal injection of corn oil (1 mouse of each strain, mock), or with a single intraperitoneal injection of corn oil containing 20 nmol/g body weight DMBA (2 mice of each strain). Mice were sacrificed 1 or 4 wks after treatment. Liver, lung and spleen were removed from each mouse and stored in All Protect Tissue Reagent (Qiagen). The mouse kidney cell lines (+/+ P22, -/- P22, -/- cl 6 P20, -/- DMBA-surviving culture P22) were treated with DMBA as described above. Total genomic DNAs were isolated using DNeasy Blood and Tissue (Qiagen) and total protein was prepared by RIPA buffer lysis. Genomic DNA samples were analyzed for exome sequences at EdgeBio using Illumina HiSeq 2000, using the Agilent SureSelect Mouse All Exon Kit. More information about the kit can be found at this link: http://www.genomics.agilent.com/CollectionSubpage.aspx?PageType=Product&SubPageType=ProductData&PageID=2478 and http://www.chem.agilent.com/Library/datasheets/Public/5990-7235en_lo.pdf. The kit results in a ~50 Mb capture region covering ~221,784 exons and 24,306 genes. C57BL/6 (B6) DNA served as reference genome sequence. 

### Exome sequence data analysis

 The small insertions and deletions (indels) identified from exome sequencing data using the GATK package were further filtered to remove any indel with low mapping quality (MQ<10), indel calling score (QUAL<10) or reads depth (DP<10). For all the exome data analysis, only indels passing the above filtering step were used. The indel frequency was calculated using the number of reads containing indels (reads mapped to alternative allele 1 and 2) divided by the total number of reads mapped to a specific indel location. Matlab “hist” function was used to generate histograms of indel frequency. To plot the frequency histogram, moving average of the frequency counts by Matlab “smooth” function was used to generate more smooth lines. To determine and remove common indels shared by two samples, the exact indel locations were used to cross-check between two indel summary files. To retrieve genes with indels in the coding region, RefSeq transcripts of mouse genome mm9 was used. 

 SNP sequences for each kidney and liver exome data set was analyzed to determine the number of genes containing clustered mutations. A gene containing a minimum of three single nucleotide variations was defined as a gene containing clustered mutations. The total number of genes containing clustered mutations was determined following the elimination of all genes that did not exhibit clustered exome mutations. 

## Results

### Allele copy number alterations in *Fhit* ^-/-^-derived cell lines and tissue

**Figure 1:**
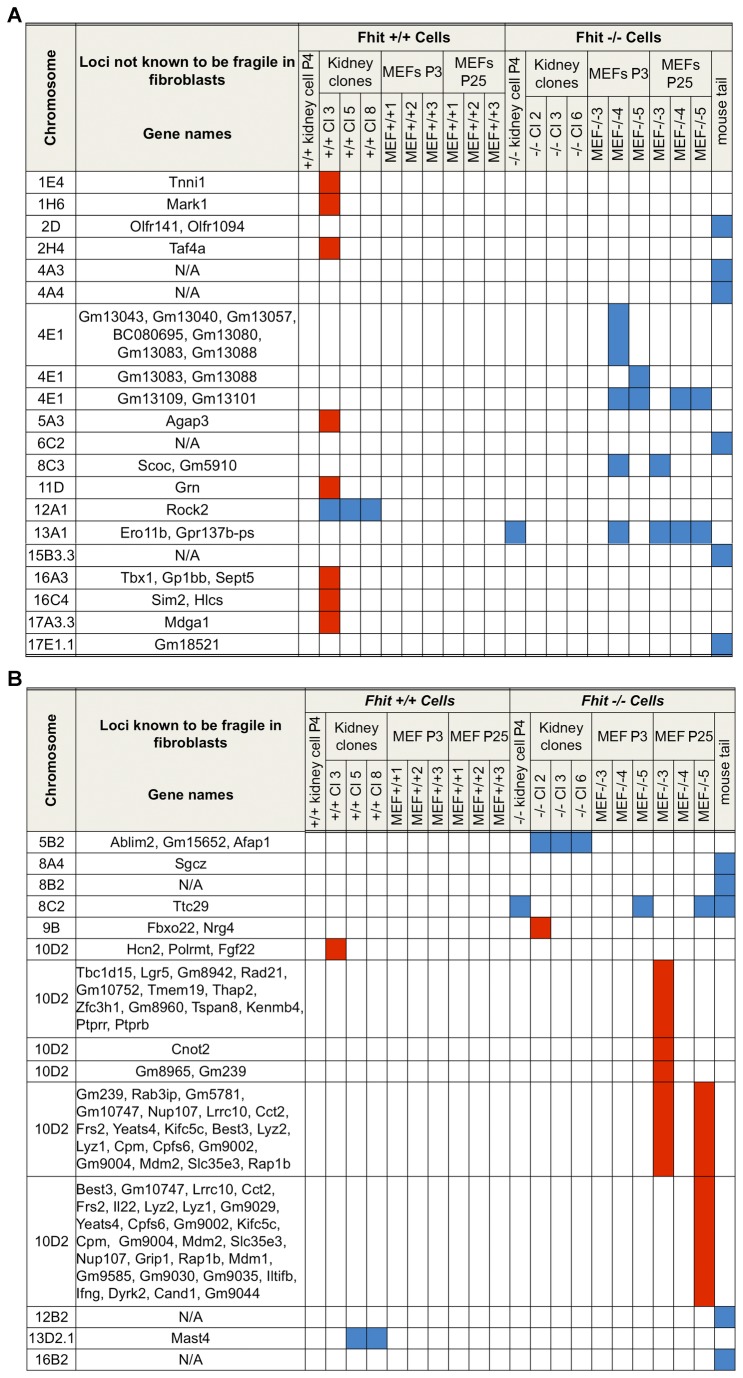
CNVs in MEFs, mouse kidney-derived cells and mouse tissue. (A) Summary of CNVs detected at chromosome loci not known to be fragile, in mouse kidney-derived cells, kidney-derived cloned cells, and pre- and post-senescence MEF cell lines derived from *+/+* and *-/*- mice, and *-/*- tail tissue, using the Affymetrix Mouse Diversity Genotype Array; CNV data for MEF cultures was previously reported [14] and is included here for comparison to the kidney culture DNAs. Blue columns indicate allele loss (fold-change range, 0.26 to 0.61), and red columns indicate gain (fold-change range, 1.51 to 1.91). For more detail, see Table S1. (B) CNVs at chromosome fragile loci. CNVs at fragile loci detected in the same mouse cell lines and tissues. Blue columns indicate allele loss (fold-change range, -0.5 to -0.83) and red columns indicate gain at specific fragile chromosome loci (fold-change range, 1.50 to 2.51).

 For examination of the genome instability induced by loss of Fhit expression, we assessed somatic CNVs, defined as DNA gains or losses spanning >10 kb, using genomic DNA isolated from *+/+* and *-/*- mouse kidney cells (at P4) and cloned cells established from mouse kidney cells (n=3), with wild type (WT) +/+ C57BL/6 (B6) tail DNA as reference. We have included the CNV results reported previously for MEF cells at P3 and P25 (n=3) and *-/*- tail DNA [14] for comparison. Figure 1A, B summarizes CNVs of >10 kb detected across the cell types tested; the genomic changes in Figure 1A represent alterations observed at genomic sites not currently known to be fragile and Figure 1B summarizes alterations observed at chromosome fragile sites, based on the reports by Djalali et al [17] for mouse fibroblasts and Hosseini et al [18] for epithelial cells. Blue in columns indicate loss of alleles, and red gain of alleles. The mean fold-change of lost alleles ranged from 0.26 to 0.83 and of gained alleles from 1.51 to 2.77 (see Table S1 for details of individual CNVs). As reported [14] previously, CNVs, mostly allele losses, occurred more frequently in *-/*- MEFs than in the comparable +/+ cells. CNVs were also detected in the genome of *-/*- weanling tail tissue, illustrating the existence of genome instability in normal tissue not related to pre-cancerous lesions and suggesting that weanling tail tissue developed from few clones. The alleles involved in most CNVs are unique to mouse kidney or MEF-derived cells. Interestingly, the established kidney cell lines and subcloned lines of both +/+ and -/- genotypes exhibit few CNVs in comparison to the -/- MEF cell lines. Most CNV loci in the tail tissue differ from those detected in the cell lines and do not correspond to known CNVs in the different mouse strains. This result suggests that specific CNVs may be selected under *in vitro* conditions and that different genome alterations contributed to survival of individual cell lines.

### The mouse kidney cell model: establishment and comparison to the MEF model

 MEF cell lines were previously established from three *+/+* and three -/- embryos and examined at early and late tissue subcultures, before and after senescence and immortalization [14]. As previously reported, CNVs were not observed in *+/+* MEF cell lines while several were detected in all *-/*- MEF cell lines (shown in Figure 1 for comparison with the CNV analysis of the kidney cell lines and clones). As noted previously, two of three post-senescence *-/*- MEF cell lines acquired amplifications within chromosome band 10D2 encompassing the murine *Mdm2* gene (Figure 1B), an oncogene frequently involved in cell transformation [20], and *Mdm2* gene amplification correlated with ~4-fold increase in *Mdm2* mRNA expression (see below for expression data). 

**Figure 2:**
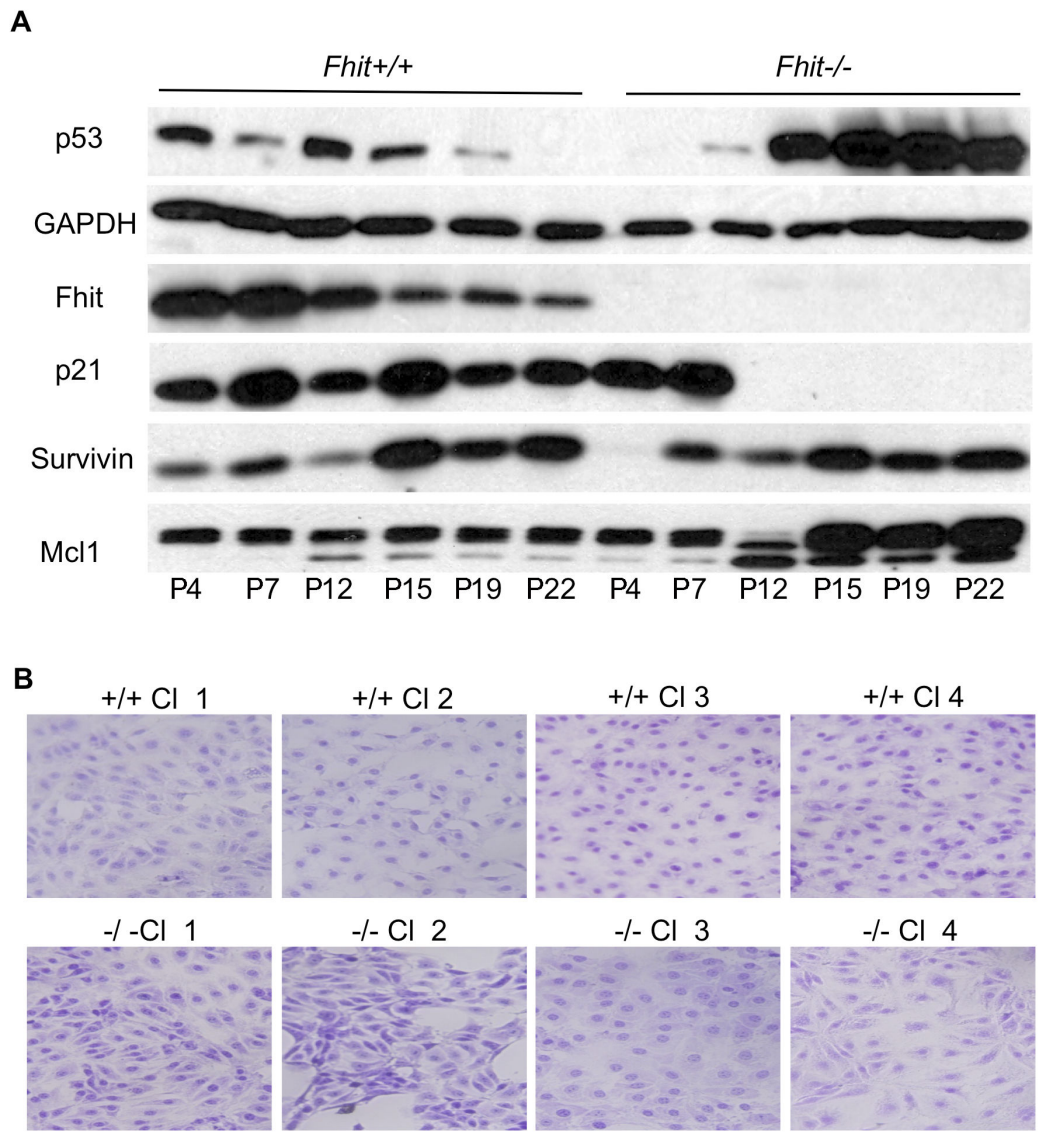
Mouse kidney cells exhibit progressive changes during *in* *vitro* culture. (A) Immunoblots for p53, p21, Survivin, Mcl1, Fhit and GAPDH expression in the mouse kidney cells with progressive culture. In *+/+* cells, Fhit expression is decreased gradually with passage progression, but capacity for apoptosis is likely maintained at late passage as shown by expression of p21. *-/*- cells undergo loss of apoptotic response with passage progression, dramatic increase of p53 in parallel with abrupt loss of p21 expression and dramatic increase of Mcl1 expression. (B) Representative micrographs of fixed, crystal biored stained +/+ and -/- kidney cell clones exhibiting morphological diversity, particularly in the -/- clones (see also Figure S1).

**Figure 3:**
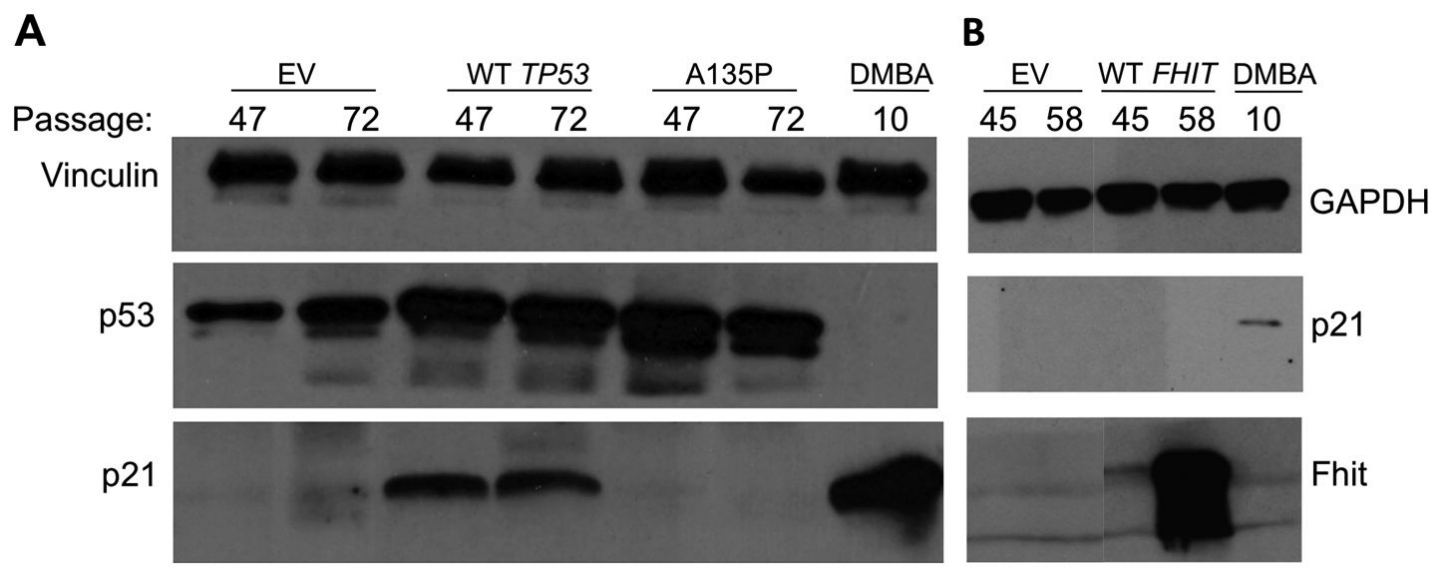
Restoration of p21 expression in late passage Fhit^-/-^ cells by WT p53 overexpression. (A) Western blot analysis of p53, p21 and vinculin in *Fhit* ^*-/-*^ mouse kidney cells transfected with either control vector (EV), WT or A135P mutant *Trp53* expression vector. The end lane shows expression of p21 in early passage *Fhit* ^*-/-*^ kidney cells treated with DMBA for 24 h. (B) Western blot analysis of p21, Fhit and GAPDH in *Fhit* ^*-/-*^ mouse kidney cells, at tissue culture passages shown across tops of gels, transfected with control (EV) or WT *FHIT* expression vector. The end lane contains lysate from early passage *Fhit* ^*-/-*^ mouse kidney cell treated 24 h with DMBA for a p21 positive control.

 Mouse kidney cells from P4 and each of eight cell lines subcloned from them at P17 were observed during establishment and proliferation at progressive tissue culture passages; Figure 2A illustrates changes in expression of survival–associated proteins from P4 to P22 of the +/+ and -/- kidney cells. The +/+ kidney cells lose expression of Fhit gradually with passage, show increasing Survivin expression and steady Mcl1 to P22 [21,22], two modulators of apoptosis. This alteration of Fhit expression levels in +/+ cells may be due to epigenetic modifications, since we have not detected allelic losses at the fragile Fra14a2 locus within the murine *Fhit* gene (Figure 1B). The most striking changes occurred in the -/- kidney cells: the p53 level increased dramatically between P7 and P12 and the *Cdkn1a* gene product, p21 [23], an important downstream p53 target, disappeared abruptly in parallel, strongly suggesting that a mutant *Trp53* gene and mutant p53 protein appeared post P7 and -/- cells carrying this mutation expanded to take over the culture by P12; though Survivin may increase slightly in parallel, Mcl1, an anti-apoptotic protein, increased in expression abruptly at P15 through P22. The absence of p21 expression is due to mutation in the p53 protein in Fhit ^-/-^ cells; when tested at P24 these cells, unlike the +/+ cells at early and late passage and -/- cells at early passage, displayed a single missense mutation, a G to C substitution, changing the amino acid at position 135 from alanine to proline; this mutation is in the DNA-binding domain of p53 and likely affects its ability to transactivate the *Cdkn1a* gene. Late passage -/- kidney cells expressing the mutant p53 protein were transfected with a WT p53 expression vector and p21 was re-expressed in the transfected cells (Figure 3). Similar over-expression of *FHIT* in the same -/- kidney cells did not result in re-expression of p21 protein (Figure 3) though it has been reported that Fhit overexpression induces expression of *Cdkn1a* mRNA [24] in H460 lung cancer cells that are negative for endogenous Fhit protein expression. This difference in results may be due to differences in cell lines used. Perhaps there is an accessory protein involved in the transcriptional activation, a protein that is missing or altered in the -/- kidney cells. Since Fhit is a cytoplasmic protein that is unlikely to act as a transcriptional activator, it is likely that its activation of *Cdkn1a* mRNA is indirect, and involves nuclear activating proteins.

 During cultivation, we observed morphological heterogeneity in the Fhit^-/-^ kidney-derived cells and isolated clonal cell populations to further examine heterogeneity in these cells; 8 cloned cell lines were established from *+/+* and -/- cells, stained with crystal biored and observed by microscopy (Figure 2B and Figure S1). The *Fhit* ^*-/-*^ clones appeared more morphologically varied than Fhit^+/+^ clones (Figure 2B, Figure S1), though there were only a few variations in CNVs. CNVs were not observed in Fhit^+/+^ mouse kidney cells at P4, though there were some allelic gains in one of the +/+ clones (Figure 1). 

### Increased clonogenicity and resistance to DMBA in late passage Fhit-deficient cells

**Figure 4:**
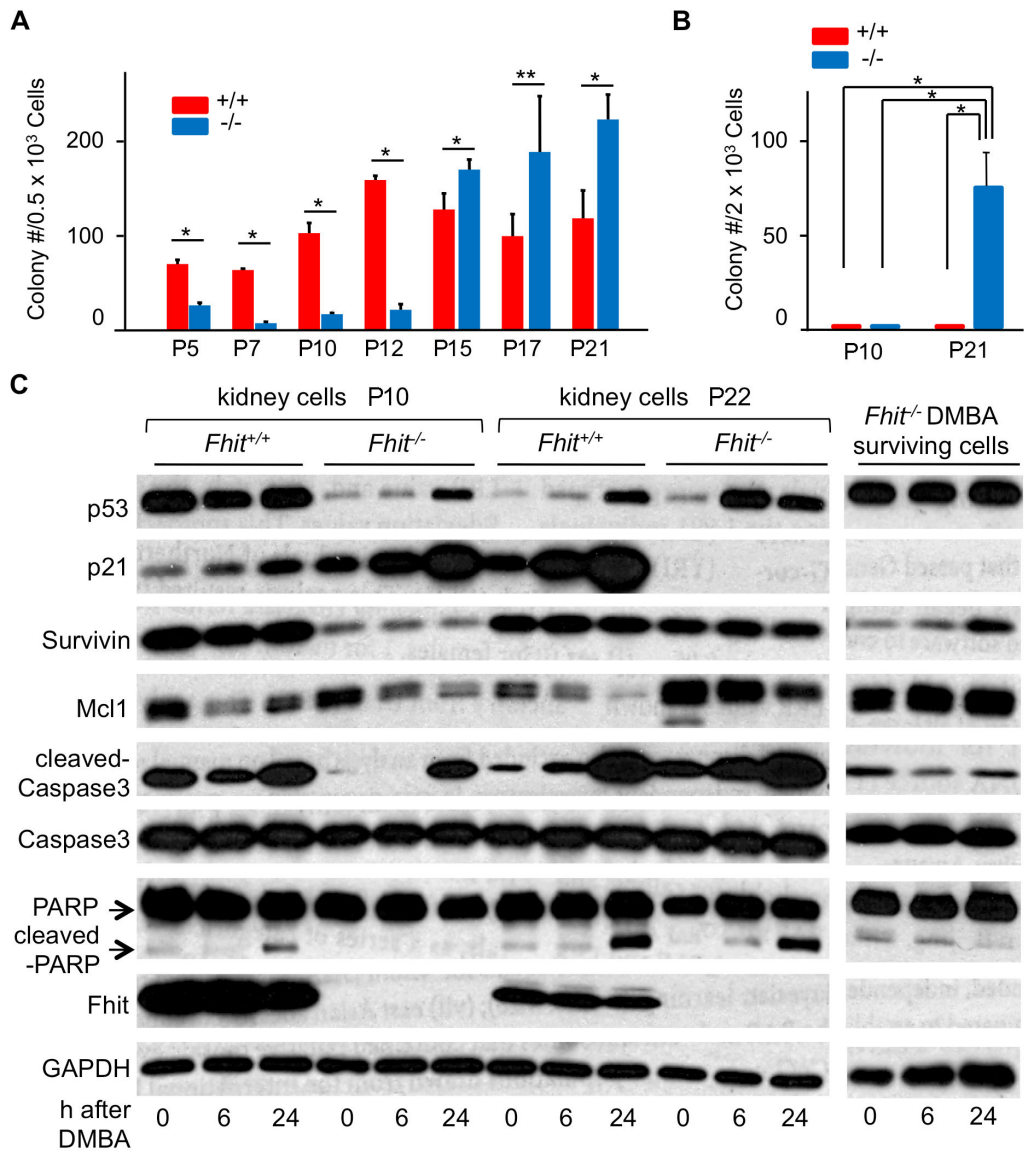
Expression of apoptosis-related proteins in *-/*- kidney cells with and without exposure to DMBA. (A) Clonogenicity of *+/+* and *-/*- kidney-derived cells with passage progression. Note the remarkable increase in colony number in -/- cells post P12 while the colony number for +/+ cells shows a steady rise to P12 and then decreases. *P <0.05; **P =0.069. (B) Clonogenicity of +/+ and -/- kidney cells after a 24 h DMBA (20 µM) treatment (at P10 and P21). *P <0.05. (C) Expression of apoptosis-associated proteins in the kidney cells before and 6 and 24 h after DMBA exposure, detected by immunoblot (at P10 and P22) (left panel). Expression of apoptosis-related proteins in -/- DMBA survivors (right panel): Note that cleaved Caspase 3 and cleaved PARP, both apoptosis markers, are markedly decreased in the DMBA-surviving cells whereas they were very similarly activated in the +/+ and -/- cells following initial DMBA exposure. In (A, B) bar graphs represent means and error bars standard deviations from at least 3 independent experiments.

**Figure 5:**
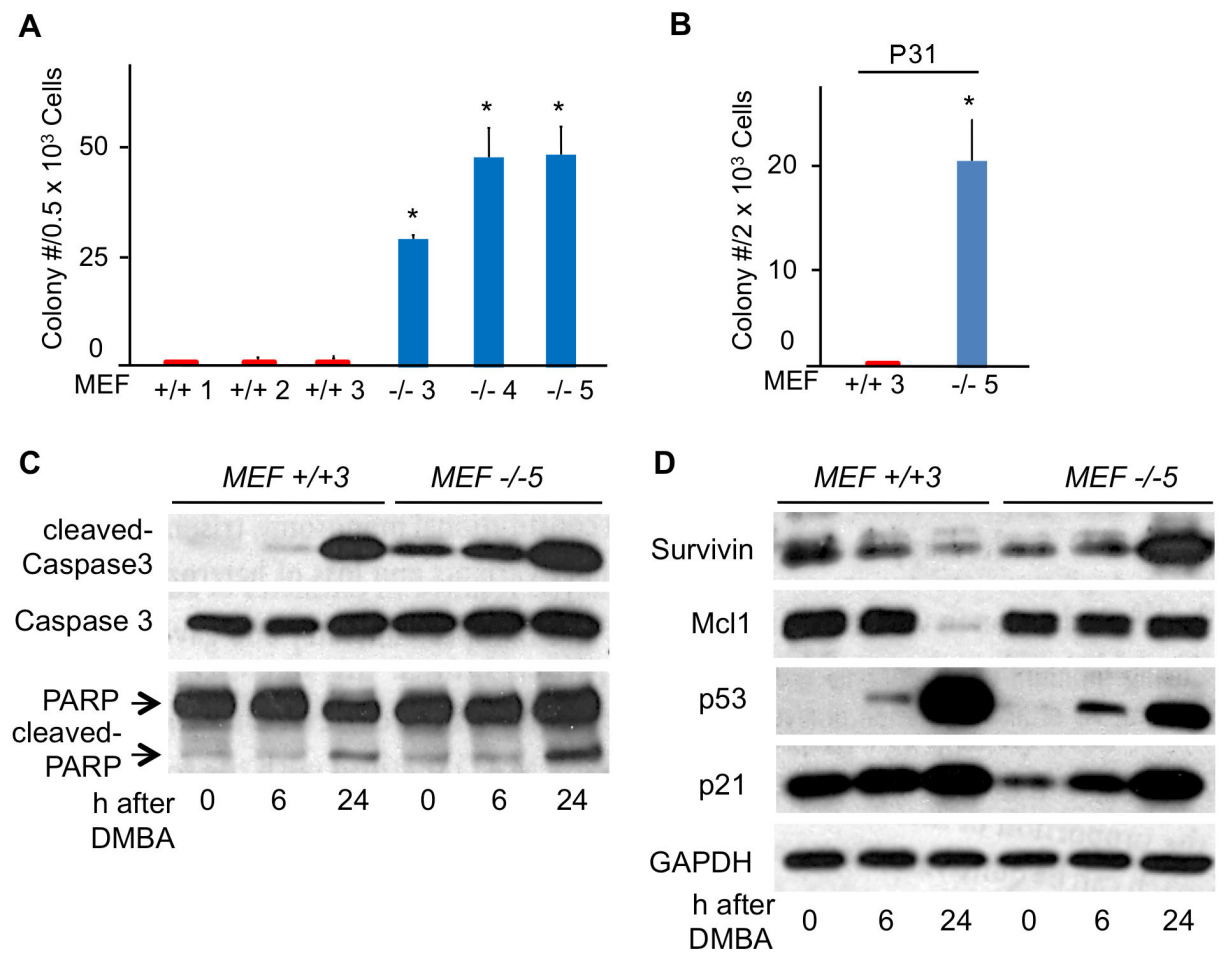
Decreased sensitivity to apoptosis-related proteins in late passage *-/*- MEF cells. (A) Clonogenicity of *+/+* and *-/*- MEFs at P30. The asterisks represent significant differences (*P <0.05) between +/+ and -/- MEFs. (B) Clonogenicity of MEF+/+ 3 and MEF-/- 5 cells after treatment with 20 µM DMBA for 24 h (P31). Bar graphs represent the means, and error bars the standard deviation from at least 3 independent experiments; (*P <0.05). (C, D) Western blots for Caspase 3, PARP, Survivin, Mcl1, p53, p21 and GAPDH expression in MEF+/+ 3 and -/- 5 cells before and 6 and 24 h after 20 µM DMBA treatment at P31. Note that although the +/+ and -/- MEFs show similar expression patterns for cleaved Caspase 3 and cleaved PARP after DMBA treatment, the MEF-/-5 cells likely survive DMBA treatment as shown in (B) because Survivin and Mcl1 are up-regulated in these cells by 24 h DMBA treatment, in contrast to the loss of these proteins at 24 h after DMBA in the MEF+/+3 cells.

 Because -/- kidney cells accumulate genome alterations due to loss of the Fhit caretaker function and showed striking changes in expression of the p53-p21 cell cycle control pathway, we were interested in how response to carcinogen exposure might differ in +/+ and -/- epithelial cells. To determine the effect of loss of Fhit expression on sequential precancerous changes *in vitro*, we assessed clonogenicity in each passage of the MEF and kidney cell lines. *-/*- kidney cells showed limited clonogenicity relative to *+/+* kidney cells at P5 to P12 (50 to 159 colonies/500 cells for *Fhit* ^*+/+*^ cells *vs* 8 to 26 colonies/500 cells for *Fhit* ^*-/-*^ cells) (Figure 4A and Figure S2A). After P15, *Fhit* ^*-/-*^ kidney cells exhibited greater clonogenicity than *Fhit* ^*+/+*^ cells (113 to 128 colonies/500 cells for *Fhit* ^*+/+*^ cells *vs* 170 to 223 colonies/500 cells for *Fhit* ^*-/-*^ cells). Similar results were observed in post-senescence MEF cells (0 to 0.25 colonies/500 cells for +/+MEF cells *vs* 30 to 50 colonies/500 cells for *-/*- MEF cells) (Figure 5A). 

 Next, we examined clonogenicity of early (P10) and late (P21) passage kidney cells treated with DMBA, an inducer of mutations and tumor initiator [15,25]. The *Fhit* ^*+/+*^ and *Fhit* ^*-/-*^ kidney cells were treated with 20 µM DMBA for 24 h and plated in 10 cm dishes. Almost no colonies were observed in *Fhit* ^*+/+*^ and *Fhit* ^*-/-*^ cells at P10 and *Fhit* ^*+/+*^ cells at P21 after DMBA treatment, while *Fhit* ^*-/-*^ cells at P21 formed 76 colonies/2000 cells (Figure 4B). Similar results were obtained in post-senescence MEF cells (0 colonies/2000 cells for MEF+/+3 cells *vs* 20 colonies/2000 cells for MEF-/-5 cells) (Figure 5B). Microscopic observation of cells 3 days after DMBA treatment showed decreased surviving cells and morphological changes in +/+ and *-/*- kidney cells at P10, *+/+* kidney cell at P21 (Figure S2B, C) and in MEF+/+3 cells (Figure S3), likely due to elimination of cells by apoptosis, as suggested by ß-galactosidase staining of some cells; *-/*- kidney cells at P21 and MEF-/-5 cells showed less morphologic change and ß-galactosidase expression [19], a marker of senescence. The results imply that the clonogenic late passage Fhit-deficient cells had acquired increased resistance to DMBA-induced apoptosis and cell cycle arrest. 

 To examine differences in expression of apoptosis related proteins, immunoblots were performed using cells before, and 6 and 24 h after exposure to 20 µM DMBA. The expression of cleaved-Caspase 3 and cleaved PARP were detected after DMBA treatment, indicating that DMBA induced apoptosis in kidney (Figure 4C) and MEF cells (Figure 5C). Apoptosis-associated proteins were similarly expressed in *-/*- kidney cells at P22 (Figure 4C) and MEF-/-5 cells (Figure 5C). Although the -/- P22 kidney cells had shown few DMBA-induced morphological changes (Figure S2C), copious cleaved-Caspase 3 was detected in the -/- P22 cells (Figure 4C). For investigation of cells that developed resistance to DMBA, we established DMBA-surviving -/- kidney cells from colonies that formed 2 wks after DMBA treatment. After expansion of these colonies, and treatment with 20 µM DMBA for 24 h, morphology and protein expression were again examined. The -/- kidney cell DMBA survivors showed neither apoptosis nor morphological changes after DMBA treatment (Figure S4). Next we examined expression of apoptosis-related protein expression after DMBA treatment. Neither the increase in expression of cleaved-Caspase 3 nor cleaved-PARP was detected in DMBA surviving *-/*- kidney cells on further DMBA treatment (Figure 4C, right panel), in agreement with absence of ß-gal staining of the cells. 

### Mechanisms of DMBA resistance differ in mouse kidney and MEF cells

 We also assessed expression of apoptosis-related proteins Mcl1, Survivin and p21 in the DMBA-treated kidney cells (Figure 4C). Mcl1 is an anti-apoptotic protein that confers resistance to Caspase-3 mediated apoptosis [22]. The level of expression of Mcl1 in *Fhit* ^*+/+*^ kidney cells at P22 and P10 was similar, while in *Fhit* ^*-/-*^ kidney cells at P22 Mcl1 expression was increased *vs* P10 cells. These expression levels were decreased in response to DMBA treatment in both +/+ and *-/*- kidney cells. Mcl1 expression levels were similar in MEF+/+3 and MEF-/-5 cells before DMBA treatment, and is still expressed in MEF-/-5 cells 24 h after DMBA treatment unlike MEF+/+3 cells (Figure 5D). The change in Survivin expression level in MEF cells after DMBA treatment was also interesting, because MEF +/+3 cells showed decreased Survivin expression, while MEF -/-5 cells showed an increase (Figure 5D). Markedly decreased expression of p21 was seen in -/- kidney cells at P22 without treatment. This expression was up-regulated after DMBA treatment in +/+ kidney cells at P10 and P22 and -/- kidney cells at P10, whereas *-/*- kidney cells at P22 exhibited negligible expression of p21 after DMBA treatment (Figure 4C). In MEF cells, induction of p21 is observed in both MEF+/+3 and MEF-/-5 cells after DMBA treatment (Figure 5D). Thus, although *-/*- kidney cells and MEF cells resistant to DMBA treatment were selected, the resistance mechanisms differed. The DMBA-surviving -/- kidney cells showed highly down-regulated cleaved Caspase 3 and reduced cleaved PARP in the complete absence of p21 expression due to mutated p53; the MEF-/- DMBA survivors instead expressed WT p53 and highly activated p21 but expressed a steady level of Mcl1 and many-fold increased Survivin during DMBA treatment. Thus, the kidney -/- cells became DMBA resistant through silencing apoptotic signals and the MEF-/- cells became resistant through activation of anti-apoptotic signals. 

### mRNA expression profile changes in *Fhit* ^+/+^ and *Fhit* ^-/-^ cells

**Figure 6:**
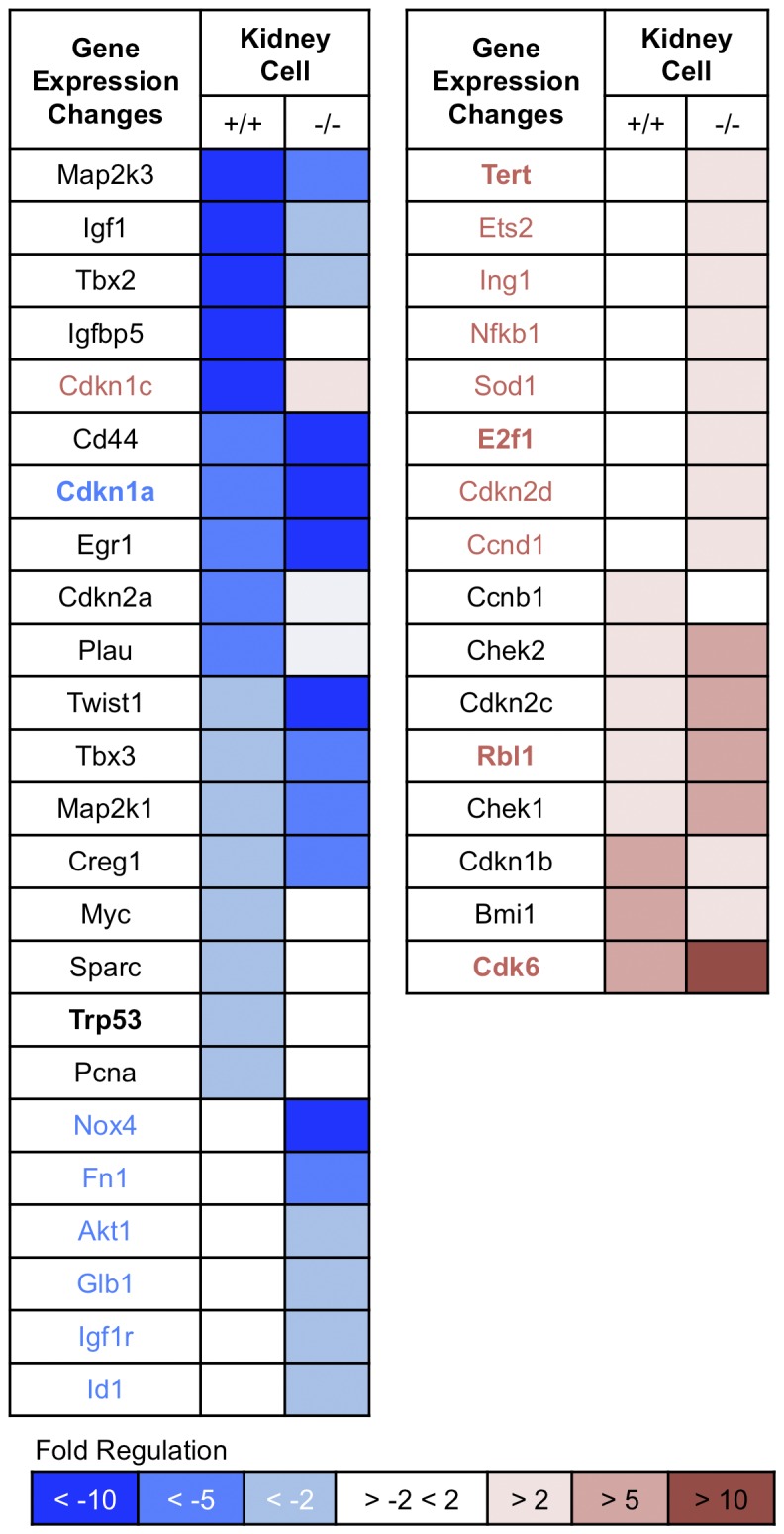
Gene expression changes in +/+ and -/- kidney cells at late *vs* early passage. mRNA expression in kidney cells at early and late passages was examined by RT² Profiler PCR Array (Mouse Cellular Senescence PCR Array, Qiagen) and fold change was calculated. Dark blue in the columns indicates >10-fold decrease of gene expression at late passage, medium blue indicates >5-fold decrease and light blue indicates >2-fold decrease. White columns indicate less than 2-fold change. Light pink indicates >2-fold increase of gene expression at late passage, medium pink indicates >5-fold increase and red indicates >10-fold increase in expression. Gene names are given in blue or red for those genes that show significant differences in expression. Gene names marked in bold indicate possibly interconnected signal pathways that are altered in *-/*- kidney cells.

 To examine gene expression changes in early and late passage kidney and MEF-derived cells, total RNA was isolated from *+/+* and *-/*- mouse kidney cells at P4 and P15 and *+/+* and *-/*- MEFs at P3 and P27 and cDNA synthesized. Senescence-associated gene expression was assessed using the RT² Profiler PCR Array (Mouse Cellular Senescence PCR Array). Data was analyzed using the Web-Based PCR Array Data Analysis program (Qiagen). Figure 6 summarizes the results for those mRNAs that showed altered expression in the kidney cells where shades of blue illustrate varying levels of reduced mRNA and shades of red varying levels of increased mRNA (complete results for the expression studies are shown in Table S2 and S3). Among down-regulated genes, *Cd44, Cdkn1a* (p21) are each down more than 10-fold in -/- kidney cells, a greater change than seen in the +/+ kidney cells; *Twist1* is also down 10-fold in -/- kidney cells *vs* 2-fold down in +/+ kidney cells. *Creg1* is down 5-fold and it has been observed that *Creg1* is decreased during immortalization [26]. *Nox4* [27,28] and *Egr1* [29] are down 10-fold and Fn *1* 5-fold. On the other hand, *Cdkn1c, Tert, Ets2, Ing1, Nfkb1, Sod1, E2f1* [30], *Cdkn2d* and *Ccnd1* are up ~2-fold in -/- kidney cells and *Chek2, Cdkn2c, Rbl1* [31,32] and *Chek1* are up ~5-fold and *Cdk6* up 10-fold in -/- kidney cells. In Figure 6, first column, gene names marked in bold indicate possibly interconnected signal pathways that are altered in *-/*- kidney cells as the basis for the enhanced proliferation, reduced apoptosis/senescence, increased clonogenicity and DMBA resistance observed. Note that *Trp53* is bold in Figure 6, to reflect the large increase in p53 protein observed in the -/- kidney cells. 

**Figure 7:**
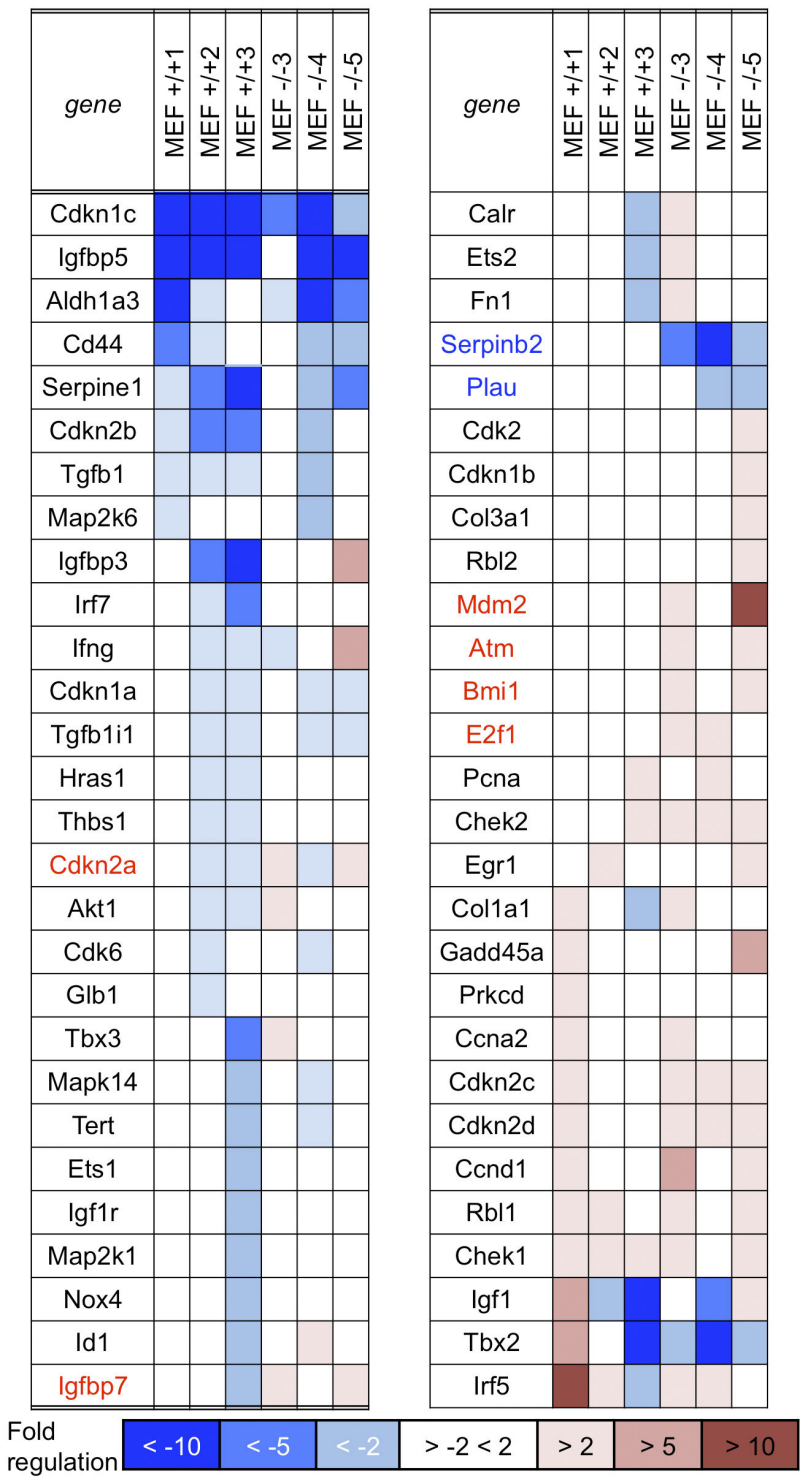
Gene expression changes in MEF cells. mRNA expression in MEF cells at early and late passages was also examined using the RT² Profiler PCR Array and fold change was calculated. Dark blue in the columns indicates >10-fold decrease of gene expression at late relative to early passage, medium blue indicates >5-fold decrease and light blue indicates >2-fold decrease. White columns indicate less than 2-fold change. Light pink indicates >2-fold increase of gene expression at late passage, medium pink indicates >5-fold increase and red indicates >10-fold increase in expression. Gene names are given in blue or red for those genes that show statistically significant differences in expression in the -/- *vs* +/+ cells in at least two of the three -/- *vs* +/+ MEF cell lines. Note the intra- and inter-strain variation in expression changes among the three +/+ and -/- MEF cell lines and the few changes in common with the kidney cell lines (E2f1, up in -/- of both cell types, Ccnd1 up in two of three -/-MEFs as well as -/- kidney); also Atm and Chek2 up in -/- lines, perhaps indicating ongoing DNA damage.

Figure 7 illustrates the varying expression patterns among both the +/+ and -/- MEF cultures. Note the increased expression of *Mdm2* in two of the -/- MEF lines, along with increased expression of *Atm, E2F1* and *Bmi1* in two MEF lines. We did not detect common mRNA expression changes in kidney and MEF cells at late *vs* early passage cells in accord with the finding that MEF and kidney cells *in vitro* selected different signal pathways for survival in culture. Exceptions are increases in expression of *E2f1* and *Ccnd1* in -/- cells of both cell types, perhaps illustrating the centrality of these proteins in control of cellular proliferation. 

### DMBA-induced mutations are increased in *Fhit* ^*-/-*^ liver tissue and kidney cells

**Figure 8:**
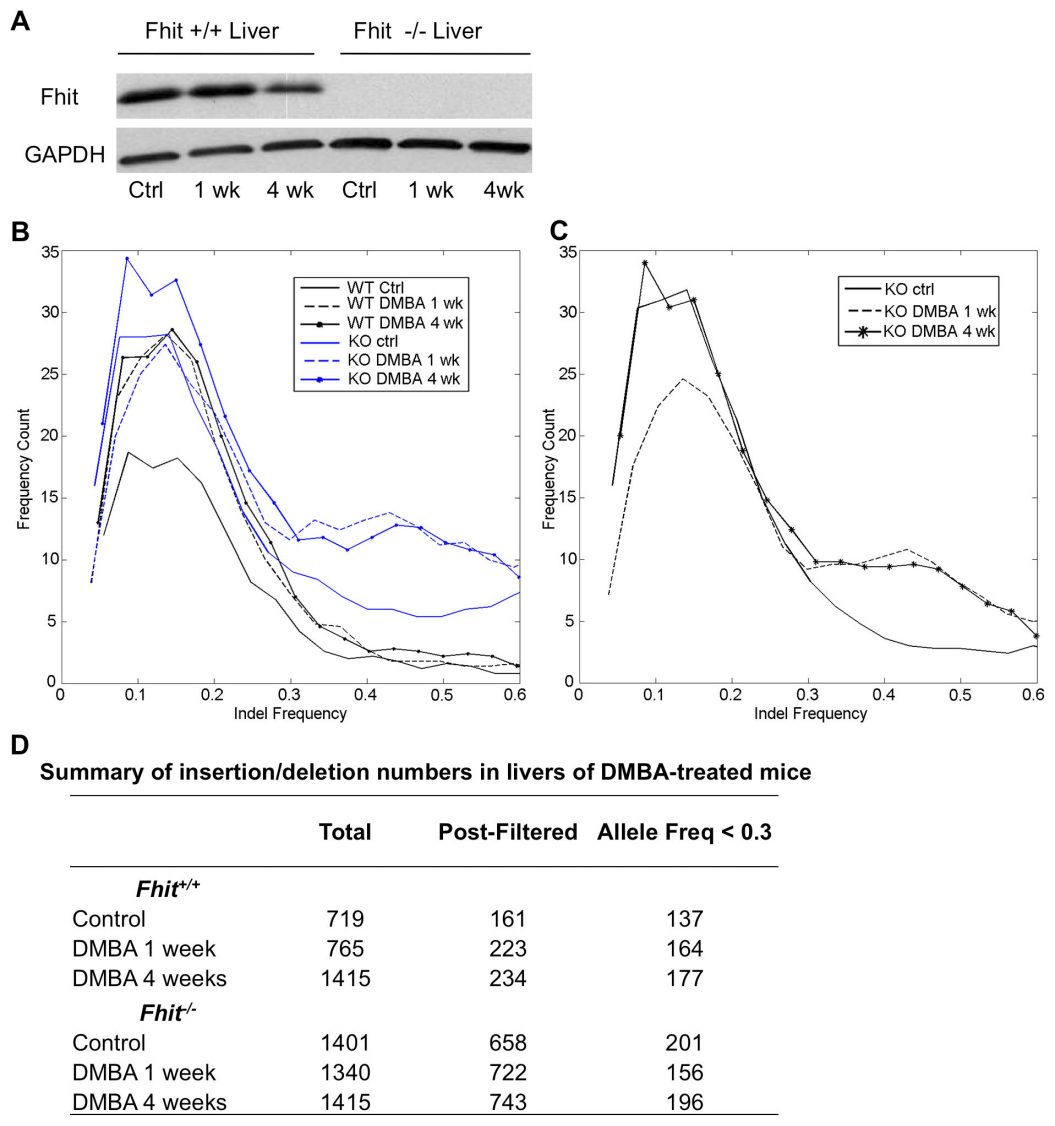
Exome insertions/deletions in mouse livers after DMBA exposure. (A) Immunoblot detection of Fhit and GAPDH expression in livers of *+/+* and *-/*- mice after DMBA treatment. (B) Histogram of spectrum of substitutions in the exomes of *+/+* mouse and *-/*- mouse liver treated with or without DMBA at indel frequencies <0.3. (C) Frequency histogram of unique indels of each -/- DNA sample. (D) Summary of insertions/deletions in the livers of control and DMBA treated mice at 1 and 4 wks post exposure. The final column represents numbers of unique indels that occurred at low frequency in liver DNAs of individual -/- and +/+ mice, possibly due to the carcinogen treatment. Indels that were common to all the mice or common to the +/+ or -/- DNAs at these low frequencies were subtracted. Note that the largest difference is between the control, untreated mice, with 201 indels in the -/- liver *vs* 137 in the +/+ liver. The control livers were from B6 mice from Jackson laboratory while the -/- livers were from -/- mice backcrossed ~9x to B6 (also Jackson lab derived many years ago) and maintained by brother-sister matings for several years. Thus the B6 control DNA would exhibit many differences relative to the -/- background genes.

 To examine the effect of Fhit-deficiency on *in vivo* carcinogen-induced mutations, the exomes of *+/+* and -/- mouse liver tissues (see Figure 8A for expression of Fhit in the tissues) were sequenced 1 wk and 4 wks following DMBA treatment; exomes of untreated *+/+* and *-/*- liver tissues were also sequenced. The total number of reads for each sample ranged from 77,650,121 to 91,060,272, and at least 98.6% of reads were aligned. The mean coverage ranged from 82X to 98X, and at least 98.1% of base pairs were covered at least 10X. After the initial filtering for small insertions and deletions (indels) based on quality scores and depth of reads (see Materials and Methods), we then subtracted all indels that were identical in both the untreated *+/+* and untreated *-/*- samples for all downstream analyses. There was a 4-fold increase in the number of indels in the -/- untreated liver tissues compared to the +/+ untreated, and a 3-fold increase in the number of indels in the DMBA-treated -/- liver tissues compared to matching +/+ liver tissues (Figure 8D). Many of the indels in the +/+ and more in the -/- samples were present at an allele frequency of greater than 0.9. Because the previous CNV analysis [14] revealed the presence of germline variants in the -/- line, we presume that the indels present at an allele frequency approaching 0.5 or 1.0 may be germline variants. Thus, to focus on somatic mutations caused by DMBA treatment or a combination of Fhit absence and DMBA exposure, only indels present in fewer than 50% of sequencing reads were considered somatic (Figure 8B and C). Furthermore, we excluded indels that were identical between untreated and DMBA-treated samples. In the untreated groups, the -/- tissues had 1.5-fold increase in the number of somatic indels compared to the +/+ tissues, whereas there was no significant difference in the number of somatic indels in the DMBA-treated +/+ *vs* -/- samples (Figure 8D). Our analysis may be an underestimate of the fold-increase in the number of somatic indels present in the -/- DNA compared to the +/+ DNA, as the majority of indels in the -/- tissues were present at an allele frequency >0.3, while the majority of indels in the +/+ tissues were present at an allele frequency <0.3. Nevertheless, the 1.5-fold increase in somatic indels in the untreated -/- confirmed *in vivo* the occurrence of mild genome instability in Fhit-negative cells.

**Figure 9:**
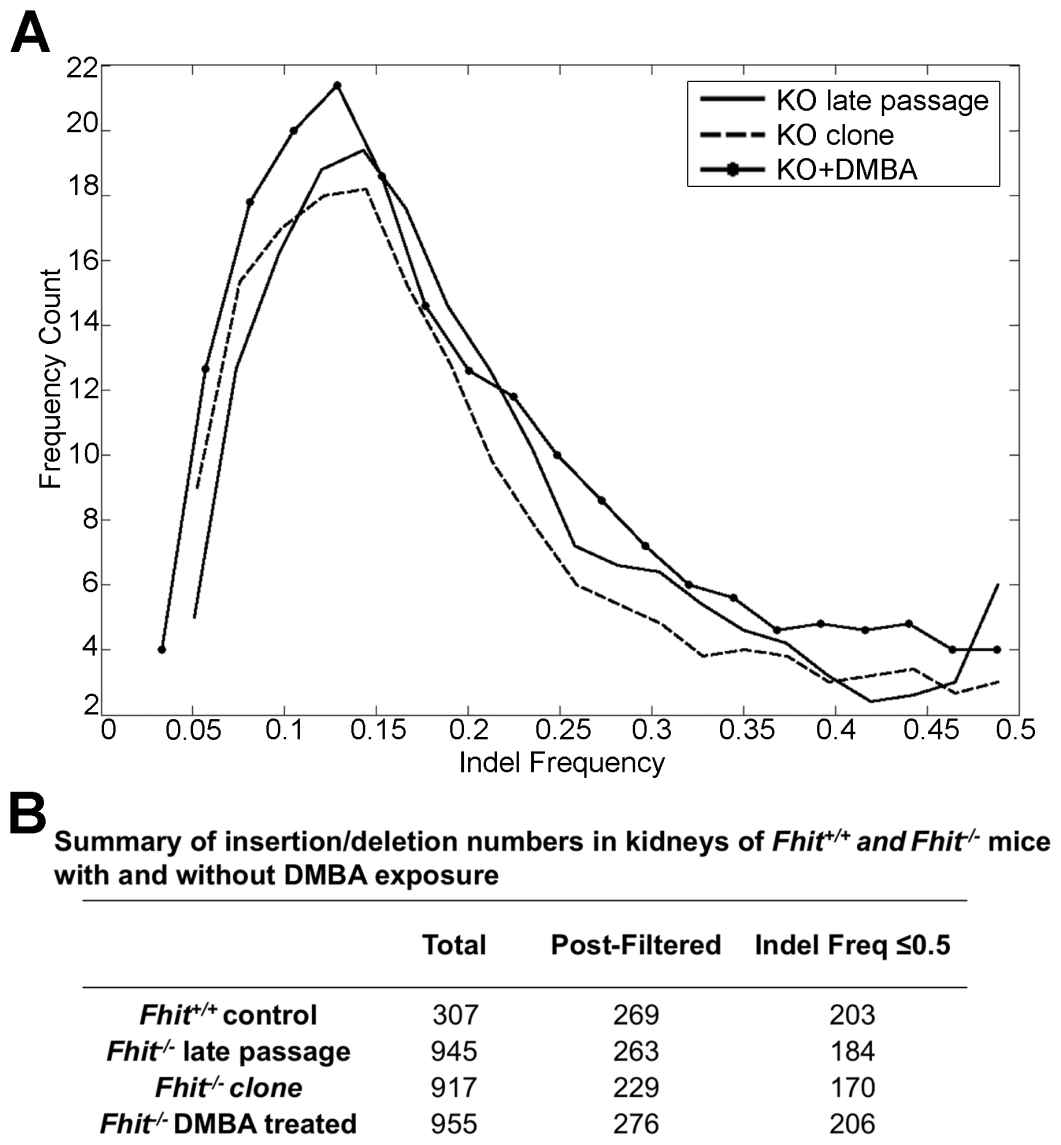
Exome insertions/deletions in mouse kidney cells with and without DMBA exposure. (A) Histogram of indel frequencies (<0.5) in -/- kidney DNAs from exome sequence data after subtraction of indels common to all of the -/- cells, -/- kidney cells at P22, -/- clone 6 kidney cells (cloned at P17 and grown for 3 more passages after cloning), and -/- kidney cells that survived treatment with DMBA at P22; (B) Summary of insertions/deletions in the kidneys of +/+ control and -/- samples with and without DMBA exposure. The final column represents numbers of unique indels that occurred at low frequency (f ≤ 0.5) in kidney DNAs of individual +/+ and -/- mice. Indels that were common to all the mice or common to the +/+ or -/- DNAs were subtracted.

 We next considered possible progressive changes 1 wk and 4 wks following DMBA treatment. In the +/+ tissues, there was an increase in the number of somatic indels (allele freq < 0.3), but no other major changes. In the -/- tissues, there was an initial decrease in the number of somatic indels with allele frequency <0.3 one week after DMBA treatment. However, at both 1 and 4 wks post-DMBA treatment, there was an increase in the number of indels with an allele frequency ranging between 0.3 and 0.6 (Figure 8B). This increase in frequency may indicate selective expansion of some liver cell populations with DMBA-induced beneficial mutations. We have also obtained exome sequences for DNA of *+/+* and -/- kidney cell lines at late passage, with and without DMBA exposure, and a figure illustrating indel frequencies in the -/- cells is shown in the histogram in Figure 9A and a summary Table in Figure 9B. A striking feature of the data in Figure 9B is that each of the -/- cell lines has accumulated ~3-fold more indels than the +/+ cells, though all were cultured for the same number of passages.

 We have analyzed the exome sequencing data in several ways, by examining overlap of detected mutations, identification of indels unique to each individual DNA sample as in Figure 8B, and for the *-/*- control liver DNA *vs* the 1 wk and 4 wk DMBA treated liver DNAs (Figure 8C). Many of the candidate mutations involve expanded or contracted minisatellite sequences and many involve deletions or insertions of a few nucleotides. See Table S4 for lists of indels in the +/+ and -/- liver DNAs with and without DMBA treatment, after removal of common indels and Table S5 for the list of indels in the mouse kidney cells that survived DMBA treatment.

## Discussion

 In hereditary cancers, genomic instability arises as a result of germline mutations in DNA repair genes; this genome instability drives cancer development in the familial cases, as predicted by the mutator hypothesis. For sporadic cancers the molecular basis of genomic instability is not known, but sequencing of the genomes of many cancers have suggested that mutations in known DNA repair genes are infrequent, possibly ruling out the mutator hypothesis for these cancers. According to a prevailing proposal, the mutation patterns observed by cancer cell large scale sequencing, documenting *TP53*, ataxia telangiectasia mutated (ATM) and cyclin-dependent kinase inhibitor 2A (*CDKN2A*; which encodes p16INK4A and p14ARF) mutations, support the oncogene-induced DNA replication stress model, which attributes genomic instability and *TP53* and *ATM* mutations to oncogene-induced DNA damage [1]. According to this proposal, genome instability in sporadic cancers results from the oncogene induced collapse of DNA replication forks, which in turn leads to DNA double strand breaks and genomic instability. 

**Figure 10:**
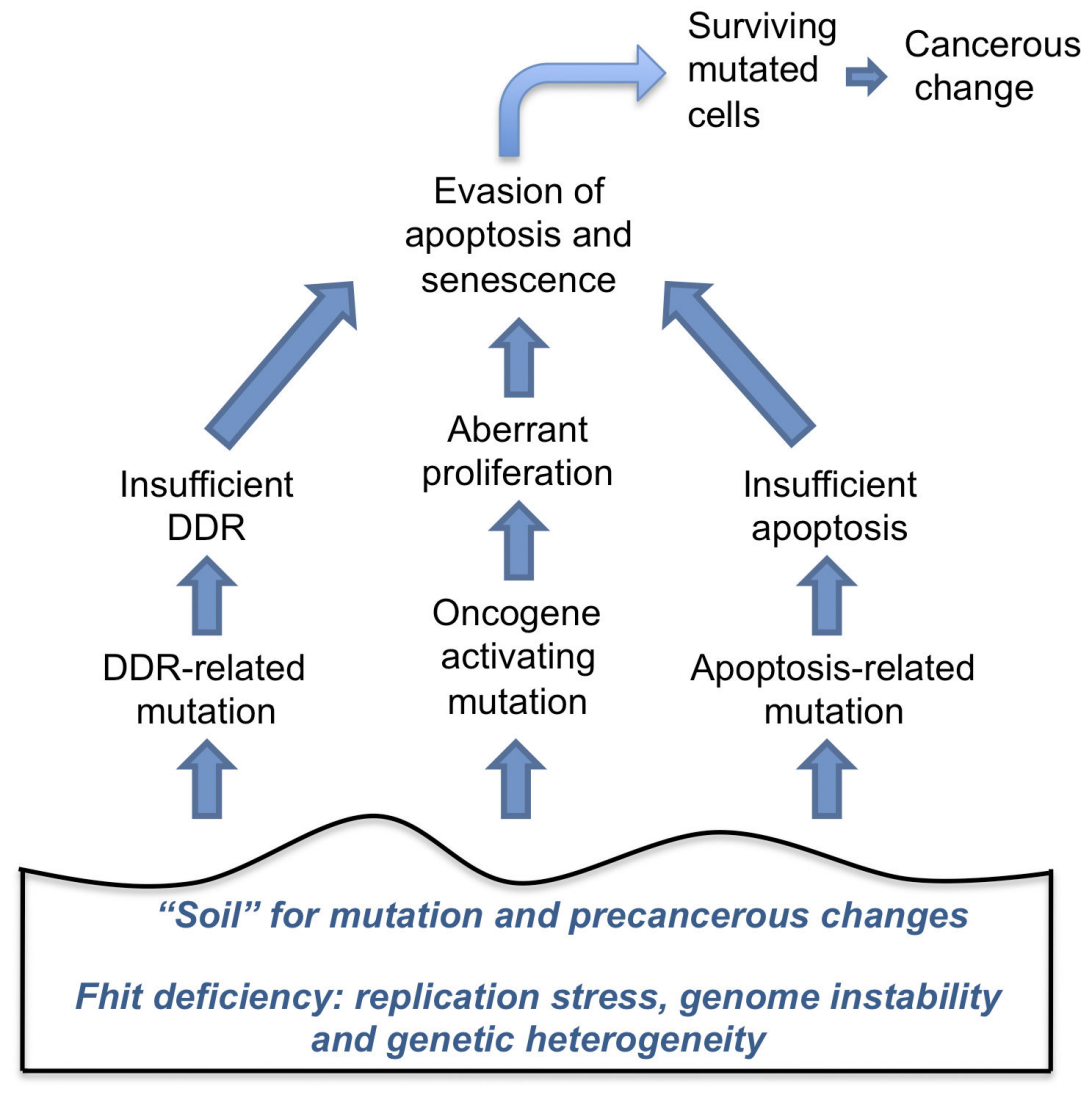
Fhit loss provides a cellular environment for selective clonal expansion. Drawing of our model summarizing the results of this study. Fhit-deficiency causes dNTP pool imbalance [14] and leads to genome instability and accumulation of mutations, under selective pressures such as tissue culture conditions or carcinogen exposure. Thus, under selection for growth, Fhit-deficient cells become impaired in the ability to eliminate cells through apoptosis and senescence. Although many cells are eliminated, some survive due to precancerous changes. Thus, the genome instability induced by Fhit loss serves as the “soil” for selective growth and progression of precancerous clones.

 The mutator hypothesis predicts that in sporadic cancers, too, mutations affecting caretaker genes should occur early in cancer development and since targeted sequencing studies to look for mutations in known DNA repair and mitotic checkpoint genes failed to identify genes that were frequently mutated in sporadic human cancers, the mutator hypothesis has fallen out of favor. Our current results suggest that Fhit might fit the prediction that ‘mutations affecting caretaker genes should occur early in cancer development’; certainly deletion of Fhit has been shown in many sequencing studies of multiple cancer types. Figure 10 presents a model that summarizes the results of this study showing that Fhit deficiency provides the ‘soil’ for mutation and precancerous changes during cancer initiation, in proliferative, senescence, apoptosis and survival pathways.

### *In vitro* growth and genome instability in *Fhit* ^-/-^ *vs* *Fhit* ^+/+^ cell lines

 As the model in Figure 10 indicates, we propose that early loss of the Fhit caretaker function sets the stage for subsequent mutation accompanied by survival pressures for expansion of clones with heterogeneous genomic, likely epigenetic, and expression changes. It is interesting that the MEF-/- cell lines show more frequent copy number alterations than the kidney cell line and derived clones. This may be deceptive since we studied only one kidney cell line from each strain, though each MEF-/- cell line shows more CNVs than the kidney -/- cell and its clones, with only a few CNVs that differ in the clones relative to the parental cell line 1 of the kidney +/+ clones has gained CNVs and the other two have just a few losses, similarly to the -/- kidney clones. This is in stark contrast to the +/+ MEFs, early and late passage, with no CNVs. These differences may be because the epithelial cells are more likely to show smaller genomic alterations and mutations and respond to survival pressures through genome and chromatin methylation, a subject for future investigation. It will be necessary to examine CNVs in more cell types from the -/- mice, including embryonic stem cells and tissues of fetal and neonatal organs as well as established cell lines to understand the patterns of CNVs in primary tissues and established cells after various environmental exposures and selective pressures. 

### Protein expression

 Through protein and mRNA expression studies, we have investigated the consequences of loss of the Fhit caretaker function as summarized in the Figure 10 model. For the immortalization and escape from senescence of the -/- kidney cell culture, it seems likely that mutation of the *Trp53* gene was the initial event that led to up-regulation of anti-apoptotic proteins and down-regulation of the executioners of apoptosis. These and associated regulatory events led to increased clonogenicity following tissue culture P12, though cleaved caspase 3 and cleaved PARP were expressed at high levels after exposure to DMBA in both the +/+ and -/- kidney cells. Clearly DMBA exposure kills many of the two cell types. A striking difference between the +/+ and -/- kidney cells was the increased frequency at which the -/- kidney cells survived DMBA exposure (Figure 4B) and were then resistant to further killing by DMBA (Figure 4C right panel), suggesting that the -/- kidney cells exhibit a higher mutation frequency following DMBA exposure, a conclusion consistent with a ‘mutator’ phenotype. It will be important to establish additional epithelial -/- cell lines to examine additional mechanisms of selection for immortality and escape from apoptosis/senescence.

*+/+* MEFs, in contrast to the +/+ kidney cells, showed very poor clonogenicity at P30 with clonogenicity of untreated -/- MEFs up to 50-fold higher and 20-fold higher after DMBA treatment (Figure 5A,B), again consistent with a mutator phenotype for the -/- MEFs. 

### mRNA Expression changes

 The Mouse Cellular Senescence RT² Profiler™ PCR Array (http://www.sabiosciences.com/rt_pcr_product/HTML/PAMM-050A.html) profiles the expression of 84 key genes involved in loss of ability of cells to divide. The cellular senescence program leads to activation of p53 and pRb signaling and to withdrawal from the cell cycle during very early tissue culture passages. The kidney cell RNAs were prepared and assessed at P4 and P15, before and after mutation of the *Trp53* gene and protein. Since the RT-PCR assay did not show a large increase in the *Trp53* mRNA level in the -/- kidney cells, though the +/+ *Trp53* level was down relative to -/-, it is possible that the mutant p53 protein was stabilized in the -/- kidney cells. Since the p21 protein was missing in -/- kidney cells (Figure 2) and its mRNA down (*Cdkn1a*, Figure 6), it is likely that the other changes such as up-modulation of *Rbl1, E2f1*, possibly *Cdk6* and *Ccnd1* followed the initial changes in the p53-p21 axis. We have studied only one +/+ and -/- kidney cell pair thus far; additional -/- kidney cell lines may follow different pathways to immortality and resistance to carcinogen-induced apoptosis.

 Interestingly, the *E2f1* mRNA level is also up in two of the MEF late passage -/- cultures. Although p53 is not mutated in the MEFs, *MDM2* is up-regulated and amplified (Figure 1) in two MEFs at late passage, thus taking a post transcriptional route to p53 inactivation, and Atm mRNA level is up in the same two MEF late passage cell lines, suggesting ongoing DNA damage. In all MEF cell lines at late passage, transcription of *Cdkn2a*/p16 is down-regulated, as expected since this step is likely involved in immortalization.

### The *in vivo* model and the mutator phenotype

 To show that the mutator phenotype induced by Fhit loss (modeled in Figure 10) could also underlie preneoplastic changes *in vivo*, we examined carcinogen-induced alterations in *-/- vs +/+* mouse livers. DMBA has been shown to cause preneoplastic changes in the liver of exposed mice as early as 4 wks after treatment: the mutation frequency after DMBA treatment was threefold higher in Gadd45a-null liver compared with wild-type liver [33]; authors concluded that lack of basal and DMBA-induced Gadd45a may result in enhanced tumorigenesis because of decreased DNA repair and increased mutation frequency, contributed by the genomic instability and loss of normal growth control in cells from Gadd45a-null mice.

 We believe that our exome sequencing data for the kidney cell DNAs and DNA of livers of DMBA exposed mice can be similarly interpreted: the livers of the -/- mice with and without DMBA showed increased numbers of exome mutations relative to the WT counterpart mouse livers. Interestingly, many of the -/- exome sequences showed clustered mutations, ie several mutations clustered in numerous individual genes as illustrated in Figure S5. This type of clustered mutations and mutation ‘showers’ have recently been shown to be functionally linked with cancer development through the action of APOBEC (apolipoprotein B mRNA-editing enzyme, catalytic polypeptide-like) cytidine deaminases [34,35], especially APOBEC3B. The mouse genome includes only one APOBEC family member gene, *APOBEC3* and we do not yet know if it is involved in development of these mutation clusters. 

 The main purpose of this study was to support the hypothesis that the Fhit loss-induced genome instability promotes, under selective conditions, the expansion of Fhit-deficient clones and the attendant mutations. The *in vitro* studies of the MEF and kidney cell lines provide evidence that Fhit-deficient cells have selective proliferative and survival advantages and the analysis of exome sequence alterations of the kidney cell and DMBA exposed liver tissue DNAs of Fhit-deficient mice showed enhanced mutation frequencies. 

 Negrini et al [1] suggested a temporal order by which the sporadic cancer hallmarks are acquired with deregulation of growth-regulating genes as the initiating event. In their model this would lead to DNA damage and DNA replication stress, which, in turn, would lead to genomic instability and selective pressure for *TP53* inactivation. Then loss of p53 function would allow evasion of cell death, while the genomic instability would provide a fertile ground for additional mutations. 

 The results of our current study suggest that, as in hereditary cancers and as modeled in Figure 10, genome instability in sporadic cancers often occurs first due to replication stress at fragile regions, followed by inactivation of Fhit, that provides continued mild genome instability, followed by the other events as in hereditary cancers.

 When the Fhit knockout mouse model was first developed and characterized, there were two noteworthy but unexplained features early in the study and another that was noted while tallying spontaneous tumors in mice between 1 and 2 years of age: first it was clear that carcinogen treatment induced many-fold more upper gastrointestinal tumors in *Fhit* ^*-/-*^ than in WT mice; secondly, some Fhit^-/-^ mice on the mixed 129XB6 background spontaneously developed Muir-Torre-like sebaceous tumors [11], that had formerly been known to occur in mismatch repair-deficient cancers. The sebaceous tumors can now be explained by the absence of the Fhit caretaker function [36] in the -/- mice and it is likely that the exquisite sensitivity to carcinogen induction of tumors is also due, in part, to loss of this function. The third feature, a relatively high rate of systemic, lethal infections among the -/- mice might [10] also be due to global genome instability if it causes mistakes in immunoglobulin or T-cell receptor rearrangements, an hypothesis not yet explored.

## Supporting Information

Figure S1 **Morphology of Fhit^+/+^ and Fhit^-/-^ kidney-derived cell clones.** (A) Representative micrographs of fixed, crystal biored stained cell lines cloned from *+/*+ and *-/*- kidney cells. (B) Immunoblots for Fhit and GAPDH expression levels in each Fhit^+/+^ cloned cell line.
(TIF)

Figure S2 **Clonogenicity and microscopic observation of DMBA-treated kidney cells at early and late passage.** (A) Colonies formed by *+/+* and *-/*- mouse kidney cells (P10 and P21). (B, C) Photographs of *+/+* and *-/*- mouse kidney cells before and 3 days after 20 µM DMBA treatment for 24 h (B, P10; C, P21). The cells were examined by light and phase-contrast microscopy after Senescence ß-gal staining.
(TIF)

Figure S3 ***Fhit*^*-/-*^ MEF cells at late passage show decreased sensitivity to apoptosis.** Photographs of MEF+/+ 3 and -/- 5 cells (P31) before and 3 days after 20 µM DMBA treatment for 24 h.
(TIF)

Figure S4 **DMBA -/- kidney cell survivors are resistant to DMBA-induced senescence.** Photographs of *-/*- DMBA survivors before, 3 and 7 days after 20 µM DMBA treatment for 24 h. The cells were examined by light and phase-contrast microscopy after Senescence ßgal staining.
(TIF)

Figure S5 **Frequency of genes with clustered point mutations in DNAs of mouse kidney cells and liver tissue.** (A) Bar graph representing comparison of numbers of genes containing clustered point mutations in -/- kidney cells that survived DMBA treatment *vs* untreated -/- and +/+ kidney cells, from exome sequence data. (B) Graphs representing comparisons of the numbers of genes containing clustered point mutations in livers of -/- and +/+ mice with and without DMBA treatment, from exome sequence data. (C) The average number of genes with clustered mutations in kidney and liver KO samples (n=6) *vs* kidney and liver WT samples (n= 4) was analyzed in a 2-tailed, unpaired Student’s t-test (P=0.000002).
(TIF)

Table S1 **Complete list of copy number variations in *Fhit*^*+/+*^ and *Fhit*^*-/-*^ cells and *Fhit*^*-/-*^ mouse tails.**
(DOCX)

Table S2 **Detailed changes in expression of the Senescence Array genes in mouse kidney cells at early and late passages (related to Figure 6).** Changes in gene expression between *+/+* and *-/*- mouse kidney cells were determined *via* relative fold-changes in the mRNA levels assayed by RT-qPCR amplification. Fold-changes were calculated by comparing the 2^-^ΔC_t_ values in *+/+* mouse kidney mRNAs at P4 to *+/+* and *-/*- mouse kidney mRNAs at P15. Fold-change values >1 indicate positive or up-regulation. Fold-change values <1 indicate negative or down-regulation. Fold-changes >2 are indicated in red; fold-change values <0.5 are indicated in blue.
(XLSX)

Table S3 **Detailed changes in expression of the Senescence Array genes in MEF cultures at early and late passages (related to Figure 7).** Changes in gene expression between *+/+* and *-/*- MEF cultures were determined *via* relative fold-changes in the mRNAs detected by RT-qPCR assays. Fold-changes represent a comparison of the 2^-^ΔC_t_ values for *+/+* MEF mRNA levels at passage 3 with *+/+* and *-/*- MEF mRNAs at passage 27. As in Table S2 above, fold-change values >1 indicate positive or up-regulation. Fold-change values <1 indicate negative or down-regulation.
(XLSX)

Table S4 **Insertions and deletions in the +/+ and -/- mouse liver DNA, with and without DMBA treatment, related to Figure 8. **
(XLS)

Table S5 **Insertions and deletions in the -/- mouse kidney cells after DMBA treatment and after subtraction of indels common to the exome sequences of the untreated -/- kidney cells (related to Figure 9).**
(XLS)
