# Supplementary figures and images for: Fhit Deficiency-Induced Global Genome Instability Promotes Mutation and Clonal Expansion
Source: PLoS One. 2013 Nov 14;8(11):e80730. doi: 10.1371/journal.pone.0080730 (PMC3828255; doi:10.1371/journal.pone.0080730)

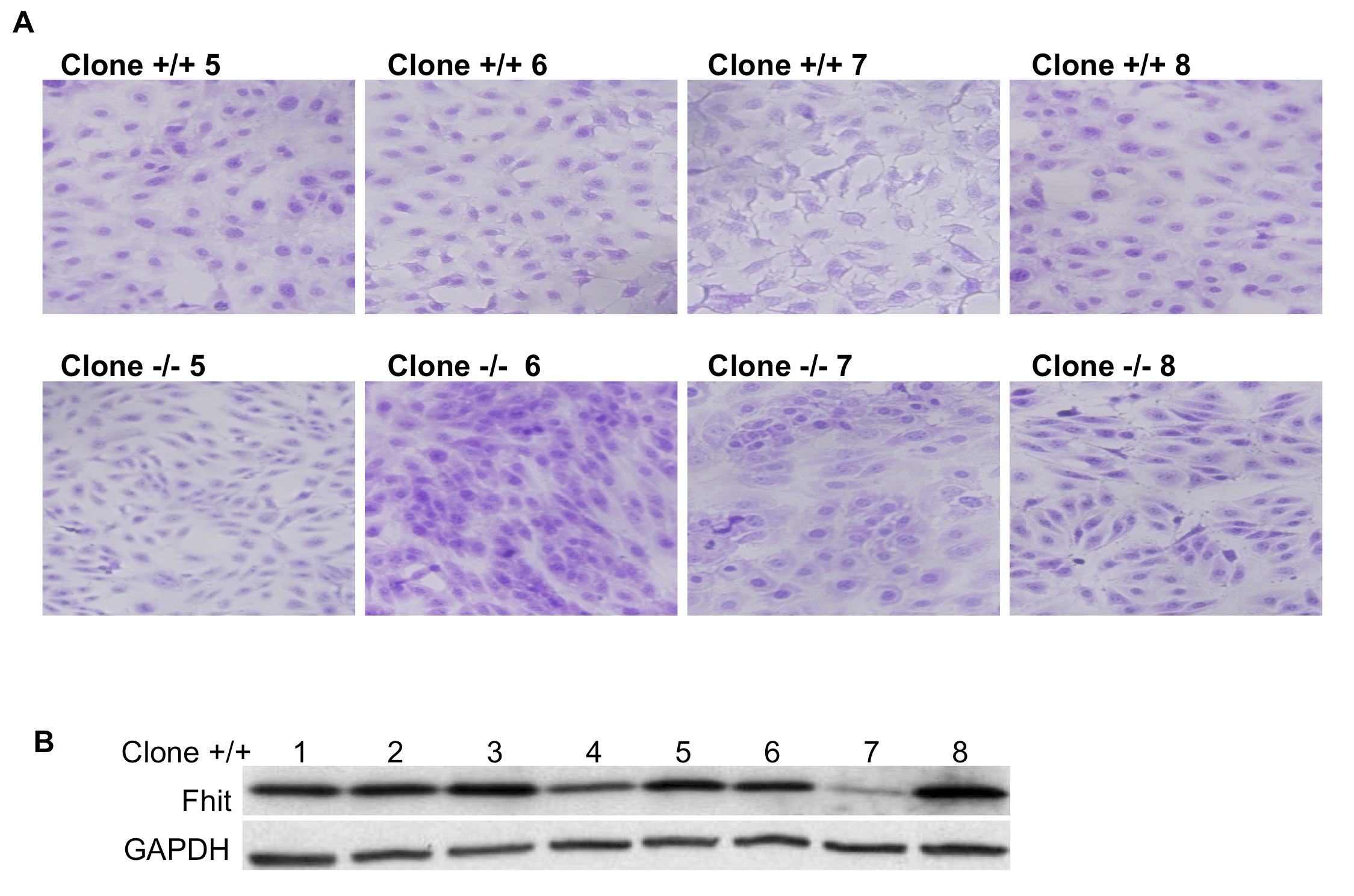

Supplement: Figure S1 — Morphology of Fhit+/+ and Fhit-/- kidney-derived cell clones. (A) Representative micrographs of fixed, crystal biored stained cell lines cloned from +/+ and -/- kidney cells. (B) Immunoblots for Fhit and GAPDH expression levels in each Fhit+/+ cloned cell line. (TIF) [file pone.0080730.s001.tif]

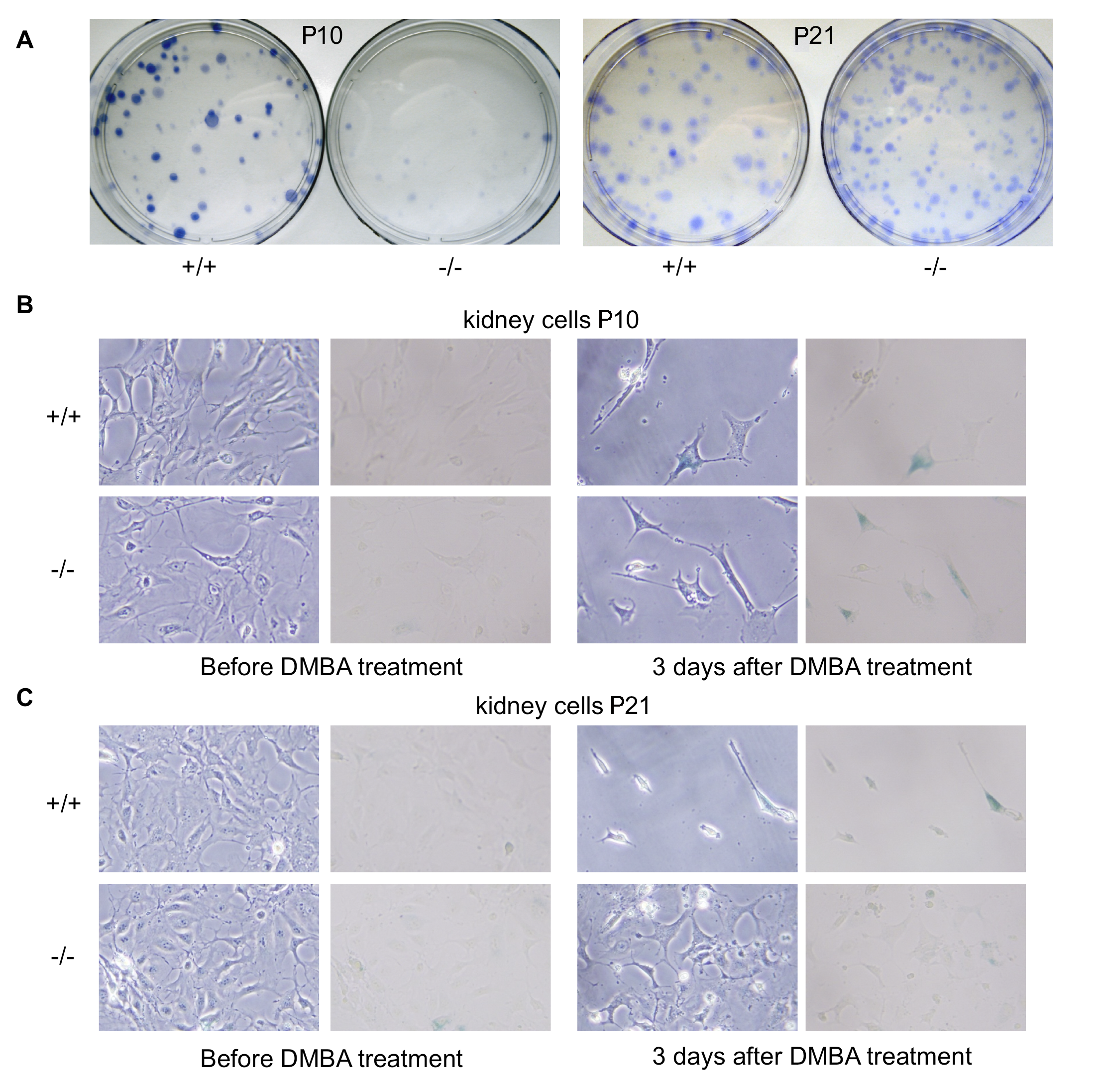

Supplement: Figure S2 — Clonogenicity and microscopic observation of DMBA-treated kidney cells at early and late passage. (A) Colonies formed by +/+ and -/- mouse kidney cells (P10 and P21). (B, C) Photographs of +/+ and -/- mouse kidney cells before and 3 days after 20 µM DMBA treatment for 24 h (B, P10; C, P21). The cells were examined by light and phase-contrast microscopy after Senescence ß-gal staining. (TIF) [file pone.0080730.s002.tif]

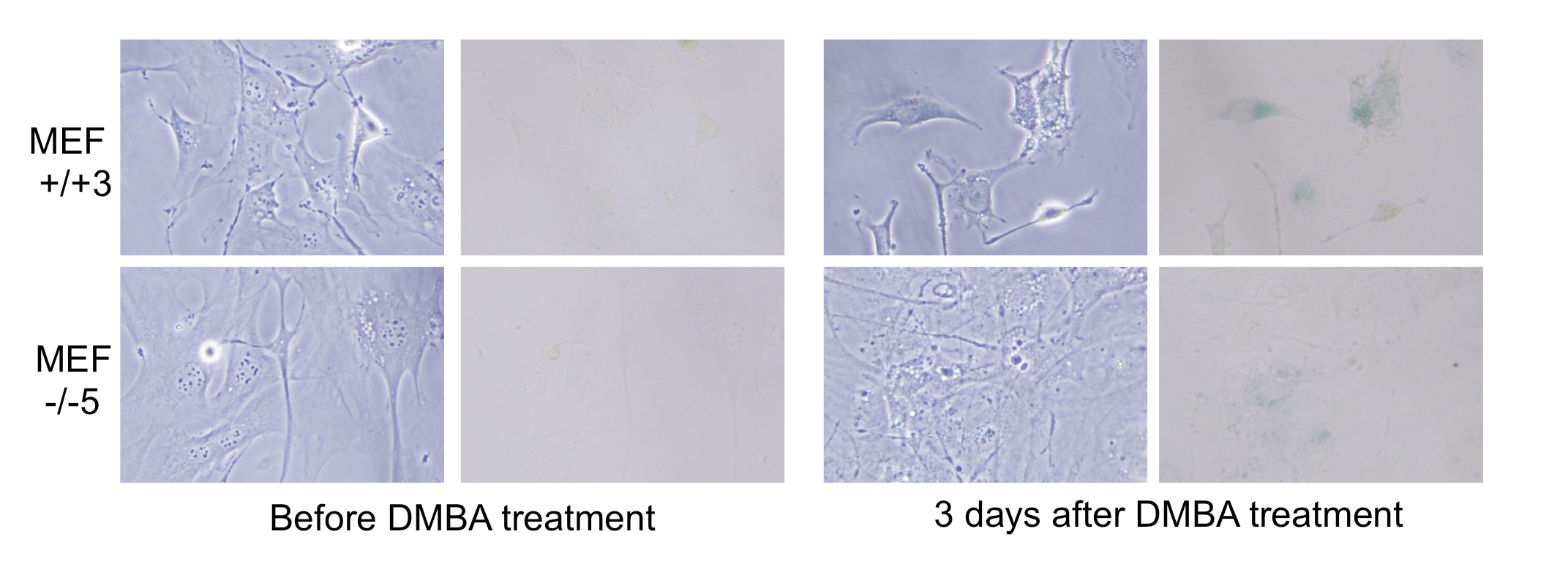

Supplement: Figure S3 — Fhit-/- MEF cells at late passage show decreased sensitivity to apoptosis. Photographs of MEF+/+ 3 and -/- 5 cells (P31) before and 3 days after 20 µM DMBA treatment for 24 h. (TIF) [file pone.0080730.s003.tif]

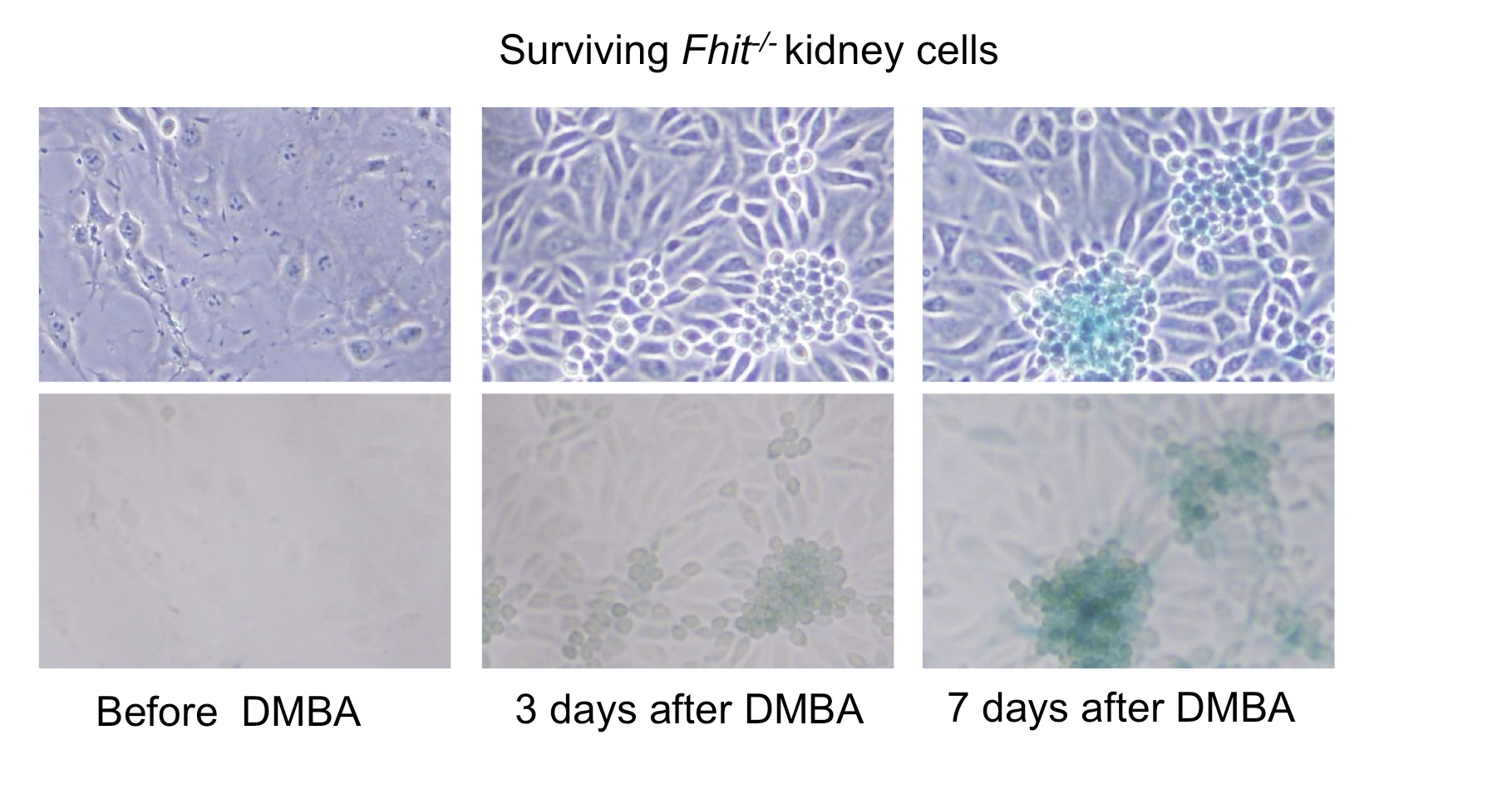

Supplement: Figure S4 — DMBA -/- kidney cell survivors are resistant to DMBA-induced senescence. Photographs of -/- DMBA survivors before, 3 and 7 days after 20 µM DMBA treatment for 24 h. The cells were examined by light and phase-contrast microscopy after Senescence ßgal staining. (TIF) [file pone.0080730.s004.tif]

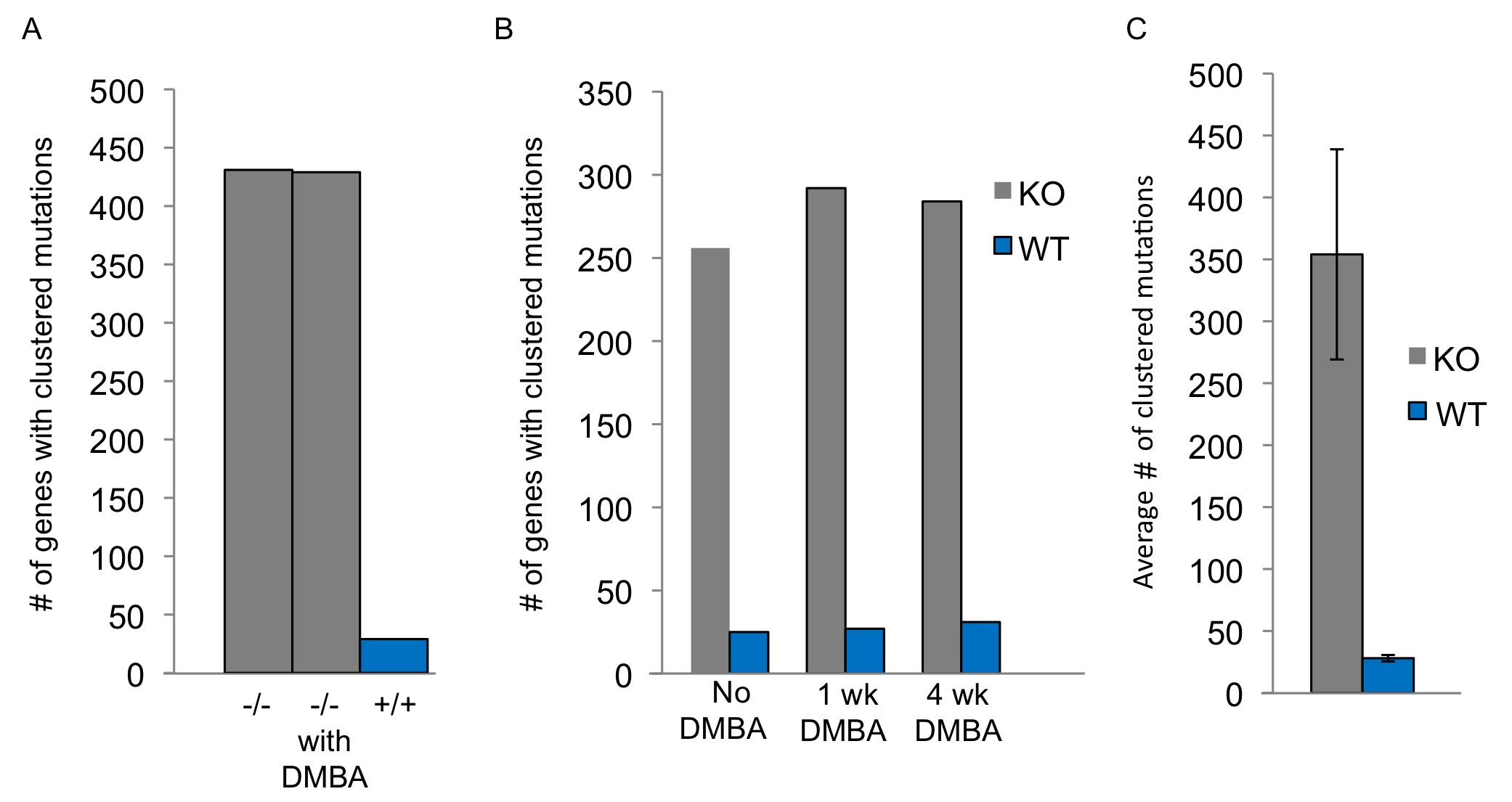

Supplement: Figure S5 — Frequency of genes with clustered point mutations in DNAs of mouse kidney cells and liver tissue. (A) Bar graph representing comparison of numbers of genes containing clustered point mutations in -/- kidney cells that survived DMBA treatment vs untreated -/- and +/+ kidney cells, from exome sequence data. (B) Graphs representing comparisons of the numbers of genes containing clustered point mutations in livers of -/- and +/+ mice with and without DMBA treatment, from exome sequence data. (C) The average number of genes with clustered mutations in kidney and liver KO samples (n=6) vs kidney and liver WT samples (n= 4) was analyzed in a 2-tailed, unpaired Student’s t-test (P=0.000002). (TIF) [file pone.0080730.s005.tif]
