# Supplementary material for: Fhit Deficiency-Induced Global Genome Instability Promotes Mutation and Clonal Expansion
Source: PLoS One. 2013 Nov 14;8(11):e80730. doi: 10.1371/journal.pone.0080730 (PMC3828255; doi:10.1371/journal.pone.0080730)
Supplement: Table S1 — Complete list of copy number variations in Fhit+/+ and Fhit-/- cells and Fhit-/- mouse tails. (DOCX) [file pone.0080730.s006.docx]

**Table S1.** Complete list of copy number variations in *Fhit^+/+^* and *Fhit^-/-^* cells and *Fhit^-/-^* mouse tail.

| ***Cell*** | ***Status*** | ***Chromosome*** | ***StartPosition*** | ***EndPosition*** | ***Gene*** | ***Size*** | ***Fold changes*** |
| --- | --- | --- | --- | --- | --- | --- | --- |
| *Fhit^+/+^* mouse kidney cell | N/A |  |  |  |  |  |  |
| Clone +/+3 | gain | 1E4 | 137685714 | 137701657 | Tnni1 | 15944 | 1.780 |
| Clone +/+3 | gain | 1H6 | 186810741 | 186870003 | Mark1 | 59263 | 1.601 |
| Clone +/+3 | gain | 2H4 | 179698714 | 179709229 | Taf4a | 10516 | 1.897 |
| Clone +/+3 | gain | 5A3 | 23958338 | 23983943 | Agap3 | 25606 | 1.814 |
| Clone +/+3 | gain | 10D2 | 79188906 | 79219650 | Hcn2, Polrmt, Fgf22 | 30745 | 1.505 |
| Clone +/+3 | gain | 11D | 102286373 | 102297662 | Grn | 11290 | 1.859 |
| Clone +/+3 | loss | 12A1 | 16879501 | 16935201 | Rock2 | 55701 | 0.311 |
| Clone +/+3 | gain | 16A3 | 18582537 | 18635467 | Tbx1, 4930588K23Rik, Gp1bb, Sept5 | 52931 | 1.507 |
| Clone +/+3 | gain | 16C4 | 94111821 | 94354808 | Sim2, Hlcs | 242988 | 1.596 |
| Clone +/+3 | gain | 17A3.3 | 29990085 | 30042940 | Mdga1 | 52856 | 1.775 |
| Clone +/+5 | loss | 12A1 | 16879501 | 16935201 | Rock2 | 55701 | 0.285 |
| Clone +/+5 | loss | 13D2.1 | 103672424 | 103813404 | Mast4 | 140981 | 0.614 |
| Clone +/+8 | loss | 12A1 | 16879501 | 16935201 | Rock2 | 55701 | 0.280 |
| Clone +/+8 | loss | 13D2.1 | 103672424 | 103797509 | Mast4 | 125086 | 0.613 |
| *Fhit^-/-^* mouse kidney cell | loss | 3A3* | 25426121 | 25445541 | Nlgn1 | 19421 | 0.389 |
| *Fhit^-/-^* mouse kidney cell | loss | 3F2.3* | 105933348 | 106103620 | Chia, Chi313, Chi314, GM6522 | 170273 | 0.618 |
| *Fhit^-/-^* mouse kidney cell | loss | 8C2 | 80806273 | 80821978 | Ttc29 | 15706 | 0.496 |
| *Fhit^-/-^* mouse kidney cell | loss | 13A1 | 12699208 | 12720056 | Ero11b, LOC100502964,Gpr137b-ps | 20849 | 0.531 |
| *Fhit^-/-^* mouse kidney cell | loss | 13A1 | 13972917 | 13987592 | Gm7446 | 14676 | 0.370 |
| *Fhit^-/-^* mouse kidney cell | loss | 16B3* | 36257364 | 36321838 | 2010005H15Rik, Stfa1, Gm4758, BC117090 | 64475 | 0.379 |
| Clone -/-2 | loss | 3A3* | 25426121 | 25445541 | Nlgn1 | 19421 | 0.496 |
| Clone -/-2 | loss | 3F2.3* | 105931765 | 106110585 | Chia, Chi313, Chi314, GM6522 | 178821 | 0.825 |
| Clone -/-2 | loss | 5B2 | 36187352 | 36327026 | Ablim2, Gm15652, Afap1 | 139675 | 0.700 |
| Clone -/-2 | gain | 9B | 55057229 | 55071236 | Fbxo22, Nrg4 | 14008 | 1.553 |
| Clone -/-2 | loss | 13A1 | 13972917 | 13987563 | Gm7446 | 14647 | 0.313 |
| Clone -/-2 | loss | 16B3* | 36257364 | 36321838 | 2010005H15Rik, Stfa1, Gm4758, BC117090 | 64475 | 0.455 |
| Clone -/-3 | loss | 3A3* | 25426121 | 25445541 | Nlgn1 | 19421 | 0.470 |
| Clone -/-3 | loss | 3F2.3* | 105931081 | 106110585 | Chia, Chi313, Chi314, GM6522 | 179505 | 0.774 |
| Clone -/-3 | loss | 5B2 | 36187352 | 36327026 | Ablim2, Gm15652, Afap1 | 139675 | 0.691 |
| Clone -/-3 | loss | 8B1.3 | 52828019 | 52894537 | Gm17993 | 66519 | 0.511 |
| Clone -/-3 | loss | 13A1 | 13972917 | 13987563 | Gm7446 | 14647 | 0.309 |
| Clone -/-3 | loss | 16B3* | 36257364 | 36321838 | 2010005H15Rik, Stfa1, Gm4758, BC117090 | 64475 | 0.476 |
| Clone -/-6 | loss | 3A3* | 25426121 | 25445541 | Nlgn1 | 19421 | 0.396 |
| Clone -/-6 | loss | 3F2.3* | 105931081 | 106103620 | Chia, Chi313, Chi314, GM6522 | 172540 | 0.731 |
| Clone -/-6 | loss | 5B2 | 36283851 | 36327026 | Afap1 | 43176 | 0.682 |
| Clone -/-6 | loss | 13A1 | 13972917 | 13987563 | Gm7446 | 14647 | 0.299 |
| Clone -/-6 | loss | 16B3* | 36257364 | 36321838 | 2010005H15Rik, Stfa1, Gm4758, BC117090 | 64475 | 0.464 |
| MEF +/+1 passage3 | N/A |  |  |  |  |  |  |
| MEF +/+2 passage3 | N/A |  |  |  |  |  |  |
| MEF +/+3 passage3 | loss | 3A3* | 25426121 | 25461978 | Nlgn1 | 35858 | 0.313 |
| MEF -/-3 passage3 | loss | 3F2.3* | 105931179 | 106110585 | Chia, Chi313, Chi314, GM6522 | 179407 | 0.611 |
| MEF -/-3 passage3 | loss | 13A1 | 13974389 | 13987563 | Gm7446 | 13175 | 0.288 |
| MEF -/-3 passage3 | loss | 16B3* | 36257364 | 36321838 | 2010005H15Rik, Stfa1, Gm4758, BC117090 | 64475 | 0.416 |
| MEF -/-4 passage3 | loss | 3A3* | 25426121 | 25445541 | Nlgn1 | 19421 | 0.340 |
| MEF -/-4 passage3 | loss | 4E1 | 143092288 | 143250513 | Gm13043, Gm13040, Gm13057, BC080695, Gm13080, LOC100044633, Gm13083, Gm13088 | 158226 | 0.575 |
| MEF -/-4 passage3 | loss | 4E1 | 143540903 | 143555553 | Gm13109, Gm13101 | 14651 | 0.389 |
| MEF -/-4 passage3 | loss | 8C3 | 85975086 | 86023239 | Scoc, Gm5910 | 48154 | 0.553 |
| MEF -/-4 passage3 | loss | 13A1 | 12699208 | 12720056 | Ero11b, LOC100502964,Gpr137b-ps | 20849 | 0.506 |
| MEF -/-4 passage3 | loss | 13A1 | 13974389 | 13987592 | Gm7446 | 13204 | 0.339 |
| MEF -/-4 passage3 | loss | 16B3* | 36257364 | 36321838 | 2010005H15Rik, Stfa1, Gm4758, BC117090 | 64475 | 0.376 |
| MEF -/-5 passage3 | loss | 3A3* | 25426121 | 25471172 | Nlgn1 | 45052 | 0.328 |
| MEF -/-5 passage3 | loss | 3F2.3* | 105933348 | 106110585 | Chia, Chi313, Chi314, GM6522 | 177238 | 0.614 |
| MEF -/-5 passage3 | loss | 4E1 | 143201660 | 143250513 | LOC100044633, Gm13083, Gm13088 | 48854 | 0.590 |
| MEF -/-5 passage3 | loss | 4E1 | 143540903 | 143555553 | Gm13109, Gm13101 | 14651 | 0.463 |
| MEF -/-5 passage3 | loss | 8C2 | 80806273 | 80834051 | Ttc29 | 27779 | 0.499 |
| MEF -/-5 passage3 | loss | 13A1 | 13974389 | 13987592 | Gm7446 | 13204 | 0.345 |
| MEF -/-5 passage3 | loss | 16B3* | 36257364 | 36321838 | 2010005H15Rik, Stfa1, Gm4758, BC117090 | 64475 | 0.403 |
| MEF +/+1 passage25 | N/A |  |  |  |  |  |  |
| MEF +/+2 passage25 | N/A |  |  |  |  |  |  |
| MEF +/+3 passage25 | loss | 3A3* | 25426121 | 25445541 | Nlgn1 | 19421 | 0.258 |
| MEF -/-3 passage25 | loss | 8C3 | 85975086 | 86023239 | Scoc, Gm5910 | 48154 | 0.568 |
| MEF -/-3 passage25 | gain | 10D2 | 114646006 | 115910232 | Tbc1d15, Gm8942, Rad21, Gm10752,Tmem19, LOC100418112, Thap2, Zfc3h1, Lgr5, A930009a15Rik, Gm8960, Tspan8, 4933416C03Rik, Ptprr, LOZC100504392, Ptprb, 1700058G18Rik, Kenmb4 | 1264227 | 2.397 |
| MEF -/-3 passage25 | gain | 10D2 | 115911553 | 116013720 | LOC100504412, Cnot2, LOC100504423 | 102168 | 2.347 |
| MEF -/-3 passage25 | gain | 10D2 | 116019245 | 116219080 | LOC100504423, Gm8965, Gm239 | 199836 | 2.769 |
| MEF -/-3 passage25 | gain | 10D2 | 116233152 | 117408874 | Gm239, Rab3ip, 4933412E12Rik, D630029K05Rik, Gm5781, Best3, Gm10747, Lrrc10, Cct2, Frs2, Yeats4, 9530003J23Rik, Lyz2, Lyz1, Cpfs6, Gm9002, Kifc5c, Gm9004, Cpm, Mdm2, Slc35e3, Nup107, LOC100504499, Rap1b | 1175723 | 2.374 |
| MEF -/-3 passage25 | loss | 13A1 | 12699132 | 12720056 | Ero11b, LOC100502964,Gpr137b-ps | 20925 | 0.500 |
| MEF -/-3 passage25 | loss | 13A1 | 13974389 | 13987592 | Gm7446 | 13204 | 0.343 |
| MEF -/-3 passage25 | loss | 16B3* | 36257364 | 36321838 | 2010005H15Rik, Stfa1, Gm4758, BC117090 | 64475 | 0.378 |
| MEF -/-4 passage25 | loss | 3A3* | 25426121 | 25445541 | Nlgn1 | 19421 | 0.302 |
| MEF -/-4 passage25 | loss | 4E1 | 143540903 | 143555553 | Gm13109, Gm13101 | 14651 | 0.385 |
| MEF -/-4 passage25 | loss | 13A1 | 12696661 | 12720056 | Ero11b, LOC100502964,Gpr137b-ps | 23396 | 0.537 |
| MEF -/-4 passage25 | loss | 13A1 | 13972917 | 13987592 | Gm7446 | 14676 | 0.363 |
| MEF -/-4 passage25 | loss | 16B3* | 36257364 | 36321838 | 2010005H15Rik, Stfa1, Gm4758, BC117090 | 64475 | 0.388 |
| MEF -/-5 passage25 | loss | 3A3* | 25426121 | 25445541 | Nlgn1 | 19421 | 0.283 |
| MEF -/-5 passage25 | loss | 3F2.3* | 105933348 | 106110585 | Chia, Chi313, Chi314, GM6522 | 177238 | 0.583 |
| MEF -/-5 passage25 | loss | 4E1 | 143540903 | 143555553 | Gm13109, Gm13101 | 14651 | 0.392 |
| MEF -/-5 passage25 | loss | 8C2 | 80806273 | 80821978 | Ttc29 | 15706 | 0.502 |
| MEF -/-5 passage25 | gain | 10D2 | 116433036 | 119040590 | Best3, Gm10747, Lrrc10, Cct2, Frs2, Yeats4, 9530003J23Rik, Lyz2, Lyz1, Cpfs6, Gm9002, Kifc5c, Gm9004, Cpm, Mdm2, Slc35e3, Nup107, LOC100504499, Rap1b, Mdm1, Il22, Gm9585, Gm9029, Gm9030, Gm9035, Iltifb, Gm9044, Ifng, Dyrk2, LOC100417929, Cand1, LOC100418236, Grip1 | 2607555 | 2.509 |
| MEF -/-5 passage25 | loss | 13A1 | 12699208 | 12720056 | Ero11b, LOC100502964,Gpr137b-ps | 20849 | 0.552 |
| MEF -/-5 passage25 | loss | 13A1 | 13972917 | 13987563 | Gm7446 | 14647 | 0.328 |
| MEF -/-5 passage25 | loss | 16B3* | 36257364 | 36321838 | 2010005H15Rik, Stfa1, Gm4758, BC117090 | 64475 | 0.389 |
| *Fhit^-/-^* Mouse tail | loss | 2D | 86646195 | 86670052 | Olfr141, Olfr1094 | 23858 | 0.540 |
| *Fhit^-/-^* Mouse tail | loss | 3F2.3* | 105933348 | 106110585 | Chia, Chi313, Chi314, GM6522 | 177238 | 0.529 |
| *Fhit^-/-^* Mouse tail | loss | 4A3 | 27102368 | 27307050 | N/A | 204683 | 0.613 |
| *Fhit^-/-^* Mouse tail | loss | 4A4 | 29717750 | 29825488 | N/A | 107739 | 0.563 |
| *Fhit^-/-^* Mouse tail | loss | 6C2 | 75342461 | 75464922 | N/A | 122462 | 0.611 |
| *Fhit^-/-^* Mouse tail | loss | 8A4 | 38773861 | 38831368 | Sgcz | 57508 | 0.565 |
| *Fhit^-/-^* Mouse tail | loss | 8B1.3 | 52224120 | 52273067 | N/A | 48948 | 0.439 |
| *Fhit^-/-^* Mouse tail | loss | 8B1.3 | 52879756 | 52943783 | N/A | 64028 | 0.545 |
| *Fhit^-/-^* Mouse tail | loss | 8B1.3 | 53127775 | 53364507 | Gm9892, Gm6463 | 236733 | 0.611 |
| *Fhit^-/-^* Mouse tail | loss | 8B2 | 55941523 | 56033681 | N/A | 92159 | 0.596 |
| *Fhit^-/-^* Mouse tail | loss | 8C2 | 80806273 | 80821978 | Ttc29 | 15706 | 0.510 |
| *Fhit^-/-^* Mouse tail | loss | 12B2 | 44361695 | 44464705 | N/A | 103011 | 0.590 |
| *Fhit^-/-^* Mouse tail | loss | 13A1 | 13972917 | 13987592 | Gm7446 | 14676 | 0.346 |
| *Fhit^-/-^* Mouse tail | loss | 15B3.3 | 49746931 | 49777457 | N/A | 30527 | 0.501 |
| *Fhit^-/-^* Mouse tail | loss | 16B2 | 27642302 | 27784650 | N/A | 142349 | 0.572 |
| *Fhit^-/-^* Mouse tail | loss | 16B3* | 36257364 | 36321838 | 2010005H15Rik, Stfa1, Gm4758, BC117090 | 64475 | 0.402 |
| *Fhit^-/-^* Mouse tail | loss | 17E1.1 | 60730307 | 60831232 | Gm18521 | 100926 | 0.580 |

*Germline CNVs not included in Figure 1 and Figure 2
